# Contrast-Optimized Basis Functions for Self-Navigated Motion Correction in Quantitative MRI

**Published:** 2024-12-27

**Authors:** Elisa Marchetto, Sebastian Flassbeck, Andrew Mao, Jakob Assländer

**Affiliations:** 1 Center for Biomedical Imaging, Dept. of Radiology, NYU School of Medicine, NY, USA; 2 Center for Advanced Imaging Innovation and Research (CAI^2^R), Dept. of Radiology, NYU School of Medicine, NY, USA; 3 Vilcek Institute of Graduate Biomedical Sciences, New York University Grossman School of Medicine, New York, New York, USA.

**Keywords:** MRF, parameter mapping, quantitative MRI, motion correction

## Abstract

**Purpose:**

The long scan times of quantitative MRI techniques make motion artifacts more likely. For MR-Fingerprinting-like approaches, this problem can be addressed with self-navigated retrospective motion correction based on reconstructions in a singular value decomposition (SVD) subspace. However, the SVD promotes high signal intensity in all tissues, which limits the contrast between tissue types and ultimately reduces the accuracy of registration. The purpose of this paper is to rotate the subspace for maximum contrast between two types of tissue and improve the accuracy of motion estimates.

**Methods:**

A subspace is derived that promotes contrasts between brain parenchyma and CSF, achieved through the generalized eigendecomposition of mean autocorrelation matrices, followed by a Gram-Schmidt process to maintain orthogonality.We tested our motion correction method on 85 scans with varying motion levels, acquired with a 3D hybrid-state sequence optimized for quantitative magnetization transfer imaging.

**Results:**

A comparative analysis shows that the contrast-optimized basis significantly improve the parenchyma-CSF contrast, leading to smoother motion estimates and reduced artifacts in the quantitative maps.

**Conclusion:**

The proposed contrast-optimized subspace improves the accuracy of the motion estimation.

## 1 | INTRODUCTION

Magnetic resonance fingerprinting (MRF) is a multiparametric quantitative magnetic resonance imaging (qMRI) approach. Its key concept is a variation of sequence parameters between TRs to maintain a transient state of the magnetization. ^1,2^

Motion-induced artifacts pose severe challenges in MRF-like experiments, whether due to physiological motion or involuntary motion due to pathological conditions. 3D acquisitions mitigate the risk of through-plane motion ^3^ - which is prohibitively difficult to correct retrospectively - but the associated long scan times make motion more likely.

Fat-navigators have previously been proposed for robust retrospective motion correction in structural brain MRI, ^4–6^ and they have successfully been implemented in a 3D MRF sequence. ^7^ However, navigator-based motion correction techniques depend on the sequence timing, as they are typically acquired during waiting periods. Moreover, even fat-selective excitation pulses can perturb the spin dynamics via the magnetization transfer effect, which can compromise quantitative measurements. An alternative approach is self-navigated motion correction, which requires no additional data as the motion parameter estimation is derived from the acquired data itself. In 3D MRF-flavored acquisitions, a self-navigated motion correction approach was previously proposed. ^8^ Kurzawski et al. reconstructed brain navigators from 7 s segments in a sub-space spanned by the truncated *singular value decomposition* (SVD) subspace. This method utilizes the low-rank nature of the underlying data and produces coefficient images which are used to extract the motion estimates using rigid registration. However, the SVD promotes high signal intensity in all types of tissue, which limits the contrast and can ultimately reduce the accuracy of the registration and extracted motion estimates.

In this work, we aim to improve the accuracy of the motion estimates by deriving a basis that maximizes the signal of the fingerprints corresponding to brain parenchyma (i.e., white and gray matter), while minimizing the signal of fingerprints from cerebrospinal fluid (CSF), therefore actively promoting the contrast-to-noise ratio.

## 2 | THEORY

The proposed contrast-optimized basis was inspired by the concept of *region-optimized virtual* (ROVir) coils, where the generalized eigendecomposition was used to maximize the signal-to-interference ratio, showing promising results in suppressing unwanted signal in different MRI applications. ^9^ We propose to use the generalized eigendecomposition to maximize the contrast-to-noise ratio between two types of tissue. This approach effectively rotates the SVD subspace, resulting in a *contrast-optimized* basis that promotes the contrast in the first and last coefficient images.

In the following, we will translate the ROVir formalism to subspace modeling. While this approach can be applied to any two types of tissue, we will outline the concept in the example of brain parenchyma and CSF and we will use simulated fingerprints to calculate the basis. First, we calculate an SVD and truncate it heuristically to the first 3 basis functions. This step ensures that the subspace covers most of the signal intensity, which minimizes artifacts from unmodeled signals.

In the second step, we project the fingerprints for brain parenchyma ${\text{s}}_{\text{b}}$ and CSF ${\text{s}}_{\text{f}}$ into the SVD subspace:

(1) $${\text{c}}_{\text{b}}={\text{U}}_{\text{SVD}}{\text{s}}_{\text{b}}$$

(2) $${\text{c}}_{\text{f}}={\text{U}}_{\text{SVD}}{\text{s}}_{\text{f}}$$

and calculate the mean autocorrelation matrices ${\text{C}}_{\text{b}}$ and ${\text{C}}_{\text{f}}$:

(3) $${\text{C}}_{\text{b}}={\text{c}}_{\text{b}}^{H}{\text{c}}_{\text{b}}$$

(4) $${\text{C}}_{\text{f}}={\text{c}}_{\text{f}}^{H}{\text{c}}_{\text{f}}.$$

The subspace that optimizes the contrast between brain parenchyma and CSF is given by the weights w that maximize

(5) $${\text{U}}_{\text{opt}}≜\frac{{\text{w}}^{H}{\text{C}}_{\text{b}}\text{w}}{{\text{w}}^{H}{\text{C}}_{\text{f}}\text{w}}$$

thus maximizing the signal fingerprints of the brain parenchyma while minimizing the signal fingerprints of the CSF. Since ${\text{C}}_{\text{b}}$ and ${\text{C}}_{\text{f}}$ are positive-semidefinite Hermitian-symmetric matrices, and ${\text{C}}_{\text{f}}$ has full rank, there is a set of eigenvalues ${\text{λ}}_{i}$ with linearly independent eigenvectors ${\text{w}}_{i}$ such that

(6) $${\text{C}}_{\text{b}}{\text{w}}_{i}=\lambda_{i}{\text{C}}_{\text{f}}{\text{w}}_{i}.$$

Here, i∈{1,2,3}. Eq. (6) can be solved by calculating the generalized eigendecomposition ^10^ for ${\text{C}}_{\text{b}}$ and ${\text{C}}_{\text{f}}$ and order the generalized eigenvalues and eigenvectors so that $\lambda_{1}\geq \lambda_{2}\geq \lambda_{3}$. Assuming normalized eigenvectors $\left(||w_{i}{||}_{2}=1\right)$, we can rotate the basis functions with

(7) $${\text{U}}_{\text{opt}}^{\left(i\right)}={\text{U}}_{\text{SVD}}^{\left(i\right)}{\text{w}}_{i}.$$

**FIGURE 1:**
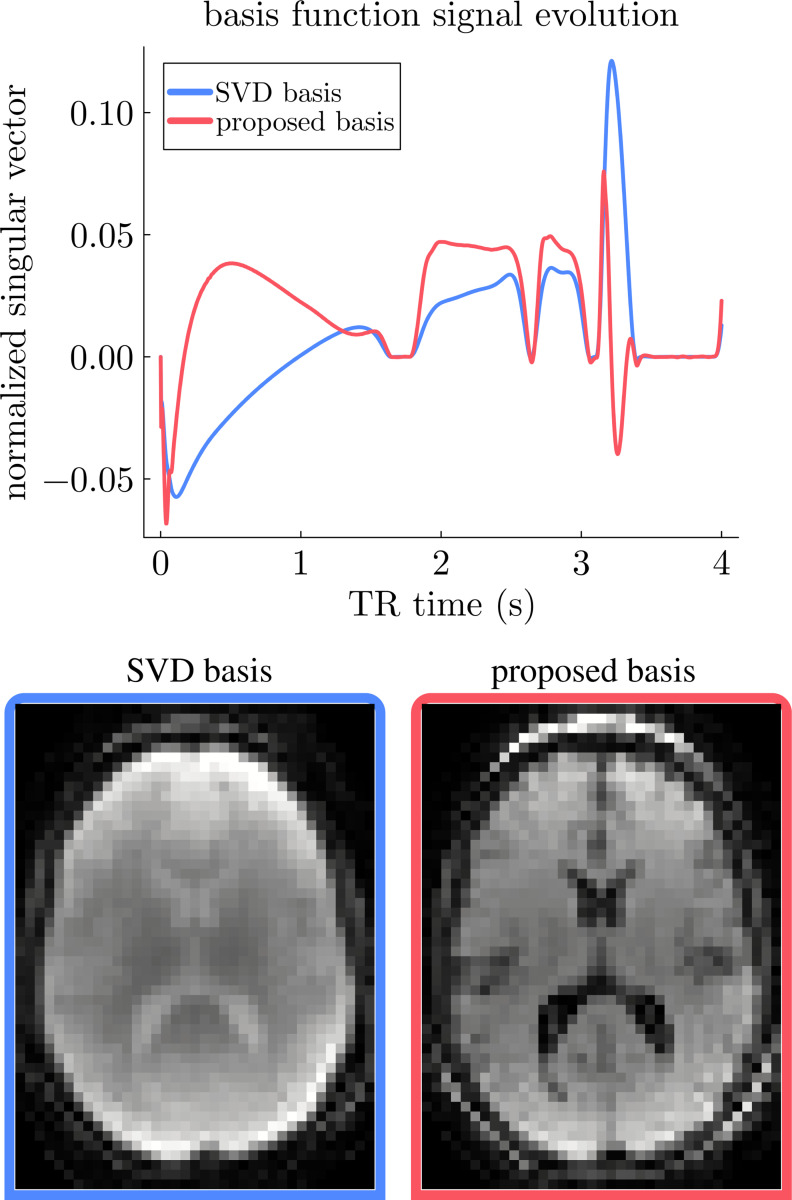
Top: Basis functions ${\text{U}}_{\text{SVD}}^{\left(1\right)}$ and ${\text{U}}_{\text{opt}}^{\left(1\right)}$, which are used to reconstruct the low-resolution images for motion estimation. The original SVD basis maximizes the overall signal, resulting in a proton-density-like contrast (bottom left). The contrast-optimized basis was designed to maximize the signal arising from the brain parenchyma while minimizing the signal arising from the CSF, resulting in improved tissue contrast (bottom right).

The rotated subspace maximizes the parenchyma and minimizes the CSF signal in the first coefficient, while the last coefficient has the opposite properties. The Gram-Schmidt approach is then applied to ensure the orthogonality between the three bases, leaving the first basis function unchanged. The resulting brain basis function ${\text{U}}_{\text{opt}}^{\left(1\right)}$ and coefficient image are shown in Fig. 1, which highlights the improved contrast between parenchyma and CSF compared to the SVD basis ${\text{U}}_{\text{SVD}}^{\left(1\right)}.$

## 3 | METHODS

### 3.1 | Data Acquisition

We acquired data from 11 healthy volunteers and 75 participants affected by mild Traumatic Brain Injury, for a total of 86 acquisitions. The participants were instructed to stay still during the scan. We used a 3 T Prisma scanner (Siemens Healthineers, Erlangen, Germany) on which we ran a 3D hybrid-state sequence ^11^ optimized for quantitative magnetization transfer (qMT) imaging. ^12^ Each RF pulse pattern is 4 s long and consists of an inversion pulse π, followed by a train of rectangular RF pulses with a varying flip angle and pulse duration and spaced 3.5 ms apart. We used six flip angle patterns, which were optimized to encode six biophysical MT parameters: $m_{0}^{s},R_{1}^{f},R_{2}^{f},R_{\text{x}},R_{1}^{s}$, and $T_{2}^{s}$. ^12^ The sequence utilizes a 3D radial koosh-ball readout trajectory with reordered golden-angle increments ^13–16^ and nominal resolution of 1 mm isotropic $\left(\left|k_{max}\right|=\pi /1\;mm\right)$, sampling 1142 spokes per cycle of the RF pattern. Each RF pattern is repeated 30 times, for a total scan time duration of 12 min. For each subject, we also acquired a 3D MP-RAGE with 1 mm isotropic resolution. Informed consent was obtained prior to the scan in accordance with our Institutional Review Board.

### 3.2 | Motion Estimation and Image Reconstruction

We aggregated all spokes from one 4 s RF cycle to reconstruct low-resolution coefficient images (4 mm isotropic) in the subspace of the SVD and the proposed contrast-optimized basis. ^17^ A total variation (TV) penalty along time was used to mitigate undersampling artifacts and noise, ^15,18^ where the associated regularization strength was chosen heuristically based on the smoothness of the motion estimates and a visual inspection of the reconstructed low-resolution images. The reconstruction problem was solved using the Alternating Directions Method of Multipliers solver. ^19^

The N=180 reconstructed low-resolution volumes were aligned separately for each flip angle pattern with the Statistical Parametric Mapping (SPM) software. ^20^ More details regarding the extraction of the motion parameters can be found in Supporting Section S1. The resulting affine matrices were then used to rotate the k-space trajectory of each 4 s block. Translations were incorporated by multiplying the k-space data with a corresponding linear phase slope.

To compensate for the limitations of the 4 s temporal resolution of motion correction, we discarded blocks before and after large jumps in the motion estimates. To this end, we calculated a *motion score* between two time points t and τ as:

(8) $$M_{t,\tau }=r_{t,\tau }+d_{t,\tau }$$

with

(9) $$r_{t,\tau }=R\sqrt{(1-\text{cos}{\left(\left|\theta_{t,\tau }\right|\right)}^{2}+\text{sin}{\left(\left|\theta_{t,\tau }\right|\right)}^{2}}$$

(10) $$d_{t,\tau }=\sqrt{{\left(x_{t}-x_{\tau }\right)}^{2}+{\left(y_{t}-y_{\tau }\right)}^{2}+{\left(z_{t}-z_{\tau }\right)}^{2}}$$

where r is the spherical distance calculated on a sphere with radius R=64mm, $\theta_{t,\tau }$ is the angle of rotation extracted from the Euler angles of the estimated rotations, and $x_{t},y_{t},z_{t}$ and $x_{\tau },y_{\tau },z_{\tau }$ are the positions of the object at times t and τ. ^5,21^ If the motion score was larger than a threshold, data from both neighboring time points were discarded in the final image reconstruction. The threshold was heuristically set at 0.75 mm, which visually resulted in the best image quality considering the trade-off between motion and undersampling artifacts. Supporting Fig. S1 provides an overview of the amount of data removed across the datasets.

Based on the motion-corrected k-space trajectory and data, the final image reconstruction was performed, also using subspace modeling. ^17,22,23^ Here, we used a subspace optimized for the conservation of the Cramér–Rao bound, ^24^ and we solved the reconstruction problem with the OptISTA algorithm. ^25^ Finally, we estimated parameter maps with a neural network-based method. ^26–28^

### 3.3 | Data Analysis

We performed a region of interest (ROI) analysis to quantify the artifact level in the parameter maps. For our pulse sequence with a radial k-space readout, motion results, among others, in noise-like artifacts. Therefore, we used the standard deviation of each quantitative parameter in each ROI as a proxy for the artifact level.

We segmented the available MP-RAGE, and used *Freesurfer* to register them to the qMT maps, focusing on the following ROIs: global white matter, the pallidum, corpus callosum, and putamen. The ROI analysis was performed on 85/86 subjects, as the MP-RAGE of one dataset was non-diagnostic due to motion artifacts.

The qMT values for each ROI were then compared against the mean *pair-wise* motion score, which is defined as:

(11) $$\bar{M}=\frac{1}{N(N-1)/2}\sum_{t=1}^{N-1}\sum_{\tau =t+1}^{N}{\text{M}}_{t,\tau }$$

The pair-wise motion score can be viewed as a proxy for the overall data inconsistency during the whole acquisition, rather than only between neighboring time points, which was proposed in Ref. 21.

## 4 | RESULTS

**FIGURE 2:**
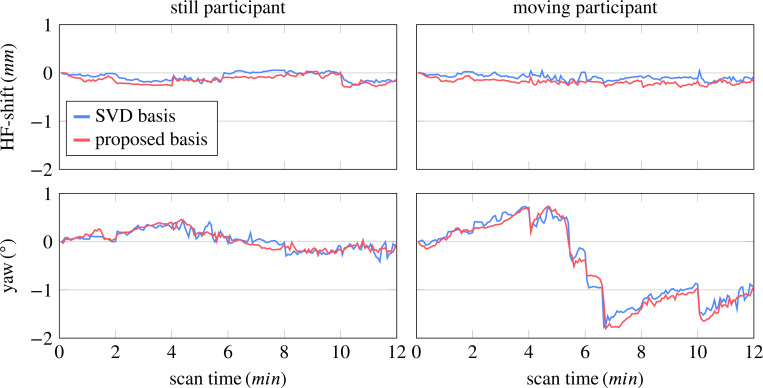
Motion estimates derived from two different acquisitions: the “still” participant moved very little during the scan, whereas the other “moving” participant moved moderately. For the still participant, the motion scores were 0.47 mm and 0.5 mm with the SVD and proposed contrast-optimized basis, respectively, and for the moving participant 1.60 mm and 1.66 mm. Here, we show one representative translation (head-foot) and one representative rotation. All 6 motion parameters can be found in the Supporting Fig. S2.

**FIGURE 3:**
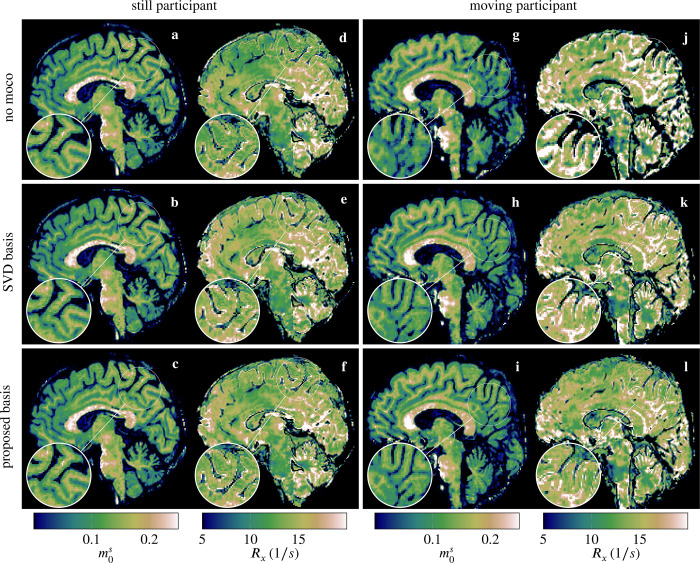
Sagittal view of $m_{0}^{s}$ and $R_{x}$ maps of a scan with virtually no motion (a–f). The motion correction with both the SVD basis (b,e) and, in particular, the proposed basis (c,f) entailed little-to-no degradation of the apparent resolution compared to a reconstruction without motion correction (a,d). In the presence of motion (“moving” participant), the parameter maps reconstructed without motion correction are degraded (g,j). Motion correction with the original SVD basis substantially reduced motion artifacts (h,k), which is further improved with the proposed contrast-optimized basis (i,l).

Fig. 2 shows estimates of two representative motion parameters for two participants. The scan on the left is virtually motion-free with translations well below 1 mm and rotations below 1°. The motion score of this case was 0.5 mm when using the contrast-optimized basis. In this example, the motion estimates based on the contrast-optimized basis appear less noisy compared to the SVD basis. The corresponding parameter maps, particularly the $R_{x}$ map (Fig. 3), and the $R_{1}^{f}$ and $R_{1}^{s}$ maps (Supporting Fig. S3) exhibit slightly reduced artifacts and enhanced details, which suggests that the contrast-optimized basis allows for accurate motion estimates. Assuming that motion is negligible in this case, we can use the reconstruction without motion correction as a reference and we observe visually a better agreement between the motion correction with the proposed basis and the reference as compared to the SVD basis (cf. Fig. 3).

In the second exemplary case - referred to as the “moving” participant - the motion score was 1.66 mm when using the proposed basis, which corresponds to the $87^{th}$ percentile of all 86 participants. The motion parameters estimated using the contrast-optimized basis appear also less noisy compared to the SVD basis. Further, we observe substantial systematic deviations (cf. right column of Fig. 2 and Supporting Fig. S2 for all motion parameters). In this case, the contrast-optimized basis elicits a substantially improved image quality in the quantitative maps, as exemplified in the $m_{0}^{s}$ maps in Fig. 3. The remaining qMT parameter maps for both participants can be found in Supporting Figs. S3 and S4.

**FIGURE 4:**
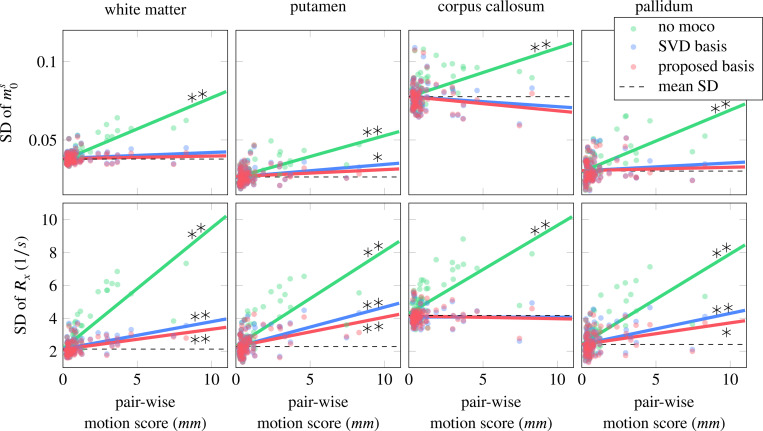
Each dot represents the standard deviation of the qMT parameter for one participant. The mean standard deviation was calculated across the three reconstruction methods. Linear regression was performed to analyze the increase of the standard deviation with increasing motion. Black stars denote slopes that differ significantly from zero (* for p-value < 0.05, ** for p-value < 0.01). The slopes of this linear regression analysis are further analyzed in Fig. 5.

**FIGURE 5:**
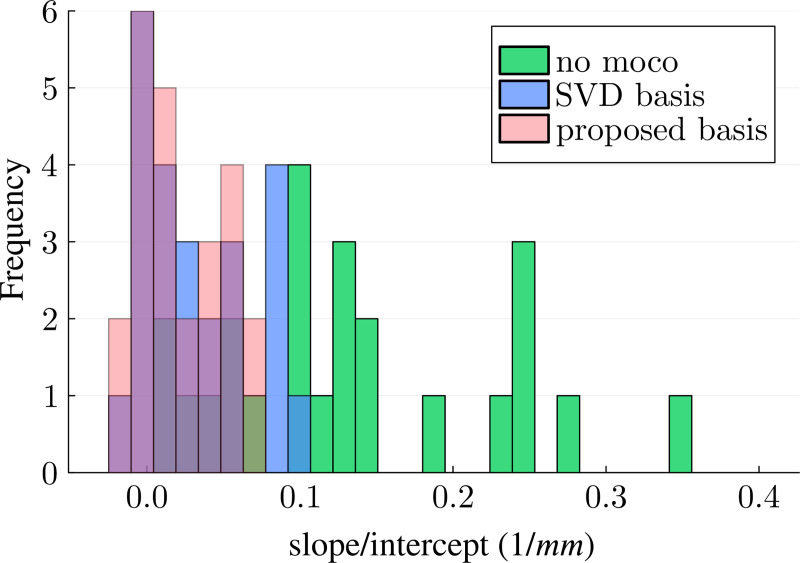
Analysis of the parameter estimates’ standard deviation in various ROIs as a function of the motion score (cf. Fig. 4). The histogram shows the slope of a linear regression, normalized by the respective intercept. This analysis pools all (6) qMT parameters and 4 ROIs. The proposed contrast-optimized basis results in smaller slopes compared to the SVD basis, indicating better motion correction.

To evaluate the performance of the motion correction across all 85 scans in our dataset, we calculated the standard deviation for the above-mentioned ROIs and analyzed them as a function of the respective pair-wise motion score (Fig. 4). Without motion correction, the standard deviation consistently increases with increasing motion, and the slope of a linear regression model differs from zero at a significance level of 0.01 for the majority parameters and ROIs (see also Supporting Fig. S5). When performing motion correction, the motion-induced parameter variability is reduced substantially, with most of the regression slopes being non-significantly different from zero, which indicates effective motion correction. Visually, the proposed basis outperforms the SVD basis in most ROIs and parameters. To confirm this improvement, we normalized the slope of each parameter and ROI by the respective intercept (no motion) and compared the three reconstructions, pooled over all qMT parameters (6) and ROIs (4) (Fig. 5). This analysis also suggests that the proposed basis outperforms the SVD basis, which is confirmed by a paired t-test, which revealed a significant reduction in the normalized slope (*p* < 0.01) when using the proposed basis instead of the SVD basis.

## 5 | DISCUSSION

We proposed to enhance the contrast-to-noise ratio between brain parenchyma and CSF by rotating the SVD basis. To this end, we used a generalized eigendecomposition, which is inspired by Region-Optimized Virtual Coils (ROVir). ^9^ We demonstrated that the increased contrast improves the motion estimates compared to an SVD basis, leading to better quality parameter maps.

The proposed basis can be used as a one-to-one replacement for a traditional SVD basis. Therefore, both approaches have the same target applications, foremost transient-state quantitative MRI techniques, such as MR-Fingerprinting, where the same spin dynamics are repeated while filling the k-space. Further, the proposed approach comes at no additional computation costs during the reconstruction.

We demonstrated that the contrast-optimized basis significantly improves the motion parameter estimation. Although we tested our method only on brain images and limited our investigation to rigid motion correction, the contrast-optimized basis can also be created for other body parts with two distinct tissue types. Like with the original SVD approach, key factors for successful implementation include 3D imaging to mitigate through-slice motion and a k-space trajectory that provides adequate coverage in each repetition of the spin dynamics, facilitating a time-segmented reconstruction. In this study, we used a koosh-ball trajectory with golden-angle increments, which repeatedly samples the center of k-space, and paired it with a TV-regularized low-resolution reconstruction to mitigate undersampling artifacts.

While the proposed motion correction substantially reduced the artifacts in the parameter maps, Figs. 3 suggests that, in cases of severe motion, the image quality is still impaired despite motion correction with either basis. This is more evident in Supporting Fig. S6, where the quantitative maps are severely degraded by the strong motion artifacts, which resulted in a motion score of 6.35 mm (motion parameters shown in Supporting Fig. S7): despite the considerable improvements obtained after motion correction (especially in the $R_{1}^{f}$ maps), the quantitative maps are still severely affected by motion artifacts. One explanation might be the inherently low temporal resolution of self-navigated motion correction. In our current implementation, each motion state is assigned every 4 s block, and improving the temporal resolution will be part of future work.

The low temporal resolution entails the assumption that no motion occurs during a 4 s block. To address this limitation, we discard data before and after strong motion. However, in cases of continuous motion, the trade-off between motion and undersampling artifacts imposes a ceiling on the motion correction performance.

## 6 | CONCLUSIONS

We propose a contrast-optimized basis function for self-navigated motion correction in quantitative MR. We utilize the generalized eigendecomposition to increase the contrast-to-noise ratio between brain tissues and CSF, which improves the accuracy of the motion estimates and, ultimately, the image quality of the quantitative parameter maps. The proposed technique does not require any sequence modifications and/or additional scan time. Consequently, it can be seamlessly integrated into various quantitative MRI methods, e.g., inversion recovery or multi-echo spin echo, where signal variations over time can be effectively captured in a low-rank subspace.

## Supplementary Material

Supplement 1

## Data Availability

The reconstruction pipeline described in Section 3.3 is implemented in *Julia v1.10*, except for the volume registration (as part of the motion estimation) which is performed using SPM in MATLAB. The codes to generate the proposed contrast-optimized basis are available on GitHub.
