# Supplementary material for: Contrast-Optimized Basis Functions for Self-Navigated Motion Correction in Quantitative MRI
Source: ArXiv. 2025 Jun 17:arXiv:2412.19552v2. Originally published 2024 Dec 27. Preprint. [Version 2] (PMC11703326)
Supplement: 1 [file NIHPP2412.19552V2-supplement-1.pdf]

SUPPORTING INFORMATION

Additional supporting information may be found in the online version of the article at the publisher's website.

Figure S1: In our acquisition scheme, we use six variable flip-angle patterns to encode six biophysical magnetization transfer (MT) parameters. Here we show the corresponding coefficient image derived using the SVD basis (a-f) and the proposed contrast-optimized basis (g-l). We observe considerable variability in the contrast between the six flip angle patterns, which can be attributed to a lack of contrast consideration in the optimization objective. In contrast, the proposed basis directly maximizes the contrast between tissues (in this case, brain parenchyma and CSF), resulting in less contrast variability. The images are reconstructed by aggregating all the radial spokes acquired during one 4 s RF cycle. More details on the reconstruction in Sec. 3.2.

Figure S2: Distribution of the amount of data removal over the 86 datasets. The data removal is based on a motion score[28] and was calculated separately for the motion estimates derived using the SVD basis[14] and the proposed contrast-optimized basis.

Figure S3: RMSE scores for simulated motion, calculated by comparing motion estimates to the ground truth. For both the SVD basis and the proposed contrast-optimized basis, we tested ten different regularization parameters λ to identify the optimal λ (star markers) for each flip-angle pattern. The proposed basis yields lower minimum RMSE scores for most flip-angle patterns across three motion patterns.

Figure S4: Estimates of moderate simulated motion (motion score of 1.77 mm). The ground truth was obtained from an online database. The RMSE score, which quantifies deviations of the motion estimates from the ground truth (Sec. 3.3.3) is 0.38 mm when using the SVD basis and 0.35 mm when using the proposed basis. Downsampling the ground-truth motion to 4 s windows provides a lower bound of 0.3 mm for the RMSE score, given the low temporal resolution.

Figure S5: Parameter maps in the presence of moderate simulated motion, complementing the m_0^s and R_x maps in Fig. 3 of the main manuscript. The underlying data was corrupted with the pattern shown in Fig. 2 and Supporting Fig. S4. The RMSE for each parameter map, compared to the reference (no motion added), is shown in the top left of the respective map. The RMSE was calculated for the entire 3D brain volume, defined by the brain mask.

Figure S6: Estimates of strong simulated motion (motion score of 6.39 mm). The ground truth was obtained from an online database. The RMSE score, which quantifies deviations of the motion estimates from the ground truth (Sec. 3.3.3) is 1.33 mm when using the SVD basis and 1.26 mm when using the proposed basis. Downsampling the ground-truth motion to 4 s windows provides a lower bound of 1 mm for the RMSE score, given the low temporal resolution.

Figure S7: Parameter maps in the presence of strong simulated motion. The underlying data was corrupted with the pattern shown in Fig. S6. The RMSE for each parameter map, compared to the reference (no motion added), is shown in the top left of the respective map. The RMSE was calculated for the entire 3D brain volume, defined by the brain mask.

Figure S8: Estimates of inherent motion using the SVD basis[14] and the proposed contrast-optimized basis. We compare here a case with minimal (0th percentile motion score) and very strong (100th percentile motion score) unintended motion. The corresponding parametric maps can be found in Fig. 5 and Supporting Figs. S9 and S10.

Figure S9: Parameter maps in the presence of minimal inherent motion (0th percentile motion score), complementing the m_0^s and R_x maps in Fig. 5 of the main manuscript. In this case, all three reconstructions have virtually identical results.

Figure S10: Parameter maps in the presence of very strong inherent motion (100th percentile motion score), complementing the m_0^s and R_x maps in Fig. 5 of the main manuscript.

Figure S11: Estimates of inherent motion using the SVD basis[14] and the proposed contrast-optimized basis. We show here a case with a small amount of unintended motion (motion score 0.65 mm, 25th percentile; no data removed). The corresponding parametric maps can be found in the Supporting Fig. S12.

Figure S12: Parameter maps in the presence of small inherent motion (25th percentile motion score). The corresponding motion parameters can be found in the Supporting Fig. S11).

Figure S13: Estimates of inherent motion using the SVD basis[14] and the proposed contrast-optimized basis. We show here a case with an average amount of unintended motion (motion score 0.91 mm, 50th percentile; 1.11% of data removed). The corresponding parametric maps can be found in the Supporting Fig. S14.

Figure S14: Parameter maps in the presence of average inherent motion (50th percentile motion score). The corresponding motion parameters can be found in the Supporting Fig. S13).

Figure S15: Estimates of inherent motion using the SVD basis[14] and the proposed contrast-optimized basis. We show here a case with a substantial amount of unintended motion (motion score 1.28 mm, 75th percentile; no data removed).

Received June 10, 2025 | Revised | Accepted
DOI:

SUPPORTING INFORMATION

Contrast-Optimized Basis Functions for Self-Navigated Motion Correction in Quantitative MRI

Elisa Marchetto*[1,2] | Sebastian Flassbeck*[1,2] | Andrew Mao[1,2,3] | Jakob Assländer[1,2]

[1]Center for Biomedical Imaging, Dept. of Radiology, NYU School of Medicine, NY, USA
[2]Center for Advanced Imaging Innovation and Research (CAI^{2}R), Dept. of Radiology, NYU School of Medicine, NY, USA
[3]Vilcek Institute of Graduate Biomedical Sciences, New York University Grossman School of Medicine, New York, New York, USA.

S1 | CONSENSUS-BASED MOTION ESTIMATES

Considering each of the six flip angle patterns separately, we registered all 30 time frames to the first time frame, repeated the process by registering them to the second time frame, and so forth. The 30 different sets of motion estimates p_i were combined into a *weighted consensus estimate* p_{cons} designed to minimize the influence of outliers:[1]

$$p_{\text{cons}}^{(k+1)} = \frac{\sum_i w_i^{(k)} p_i}{\sum_i w_i^{(k)}}.$$

The consensus estimation is an iterative process where k denotes the iteration, and the weights w_i are given by

$$w_i^{(k)} = \frac{1}{1 + \|p_i - p_{\text{cons}}^{(k)}\|_2}.$$

The iterative process is terminated when the change in p_{cons} falls below $1.8 \cdot 10^{-4}$ mm or $1.8 \cdot 10^{-4}$ rad, or after 100 iterations.

The benefit of the consensus estimates over selecting a single reference is that it mitigates the risk of poor motion estimates if the reference contains artifacts.

The rigid registration was performed using the Statistical Parametric Mapping (SPM) software with the default parameters.[2] To exclude the neck region from alignment, we used a brain mask calculated with the *Freesurfer* tool *mri_synthstrip*.[3,4]

To combine the motion estimates of each flip angle pattern, we reconstructed high-resolution (1 mm isotropic) images for each flip angle pattern after performing intra-pattern motion correction. To this end, we combined all the data of respective flip angle patterns (2 min of acquisition), used the proposed contrast-optimized basis, along with locally low-rank regularization,[5–7] solved with the OptISTA algorithm.[8] We registered the 6 volumes (one for each flip angle pattern) using *Freesurfer*'s *mri_robust_register*.[9] Since these reconstructions are generally of higher quality, we simply used the second flip angle pattern as the reference volume.

* E. Marchetto and S. Flassback contributed equally to this work.

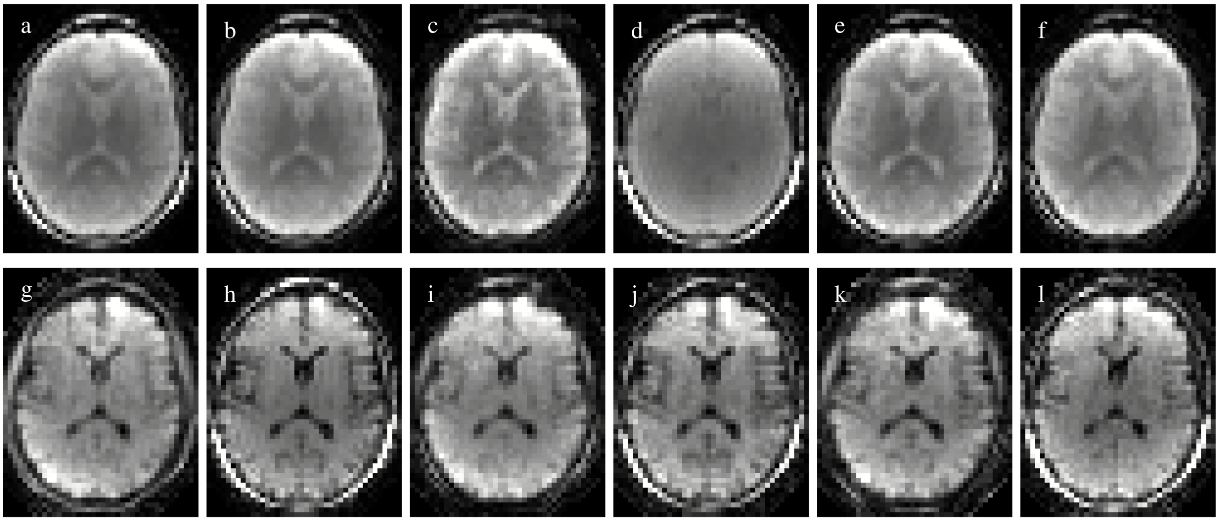

FIGURE S1 In our acquisition scheme, we use six variable flip-angle patterns to encode six biophysical magnetization transfer (MT) parameters. Here we show the corresponding coefficient image derived using the SVD basis (a-f) and the proposed contrast-optimized basis (g-l). We observe considerable variability in the contrast between the six flip angle patterns, which can be attributed to a lack of contrast consideration in the optimization objective. In contrast, the proposed basis directly maximizes the contrast between tissues (in this case, brain parenchyma and CSF), resulting in less contrast variability. The images are reconstructed by aggregating all the radial spokes acquired during one 4 s RF cycle. More details on the reconstruction in Sec. 3.2.

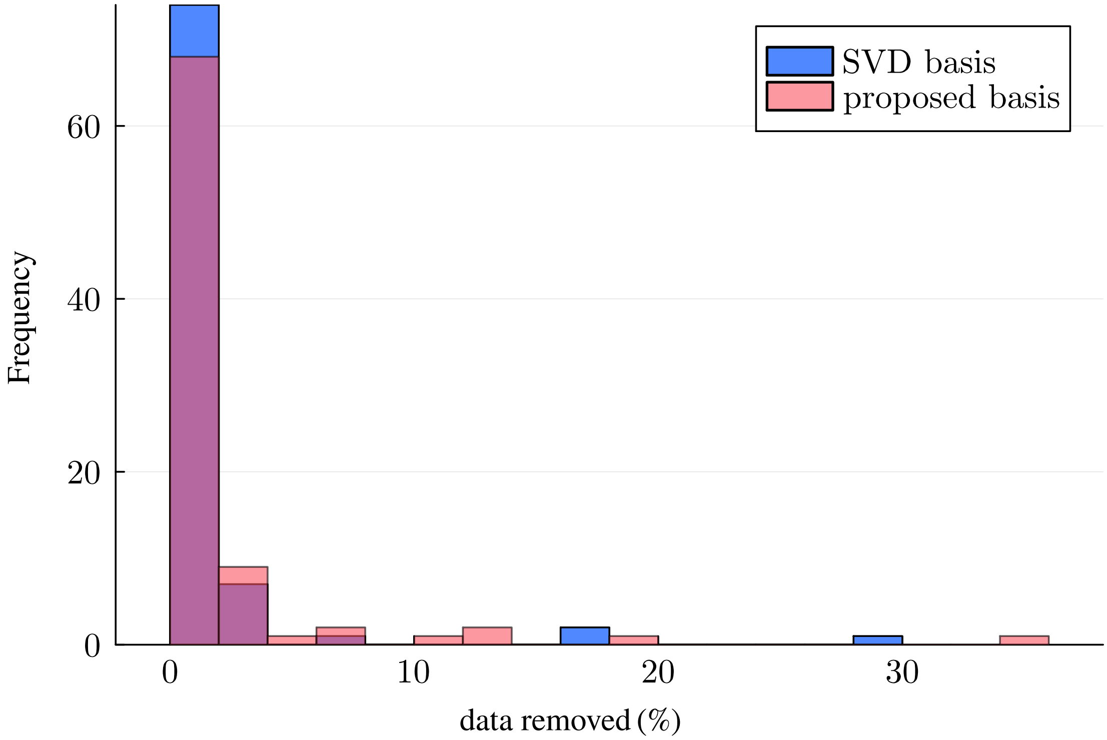

FIGURE S2 Distribution of the amount of data removal over the 86 datasets. The data removal is based on a motion score [10] and was calculated separately for the motion estimates derived using the SVD basis [11] and the proposed contrast-optimized basis.

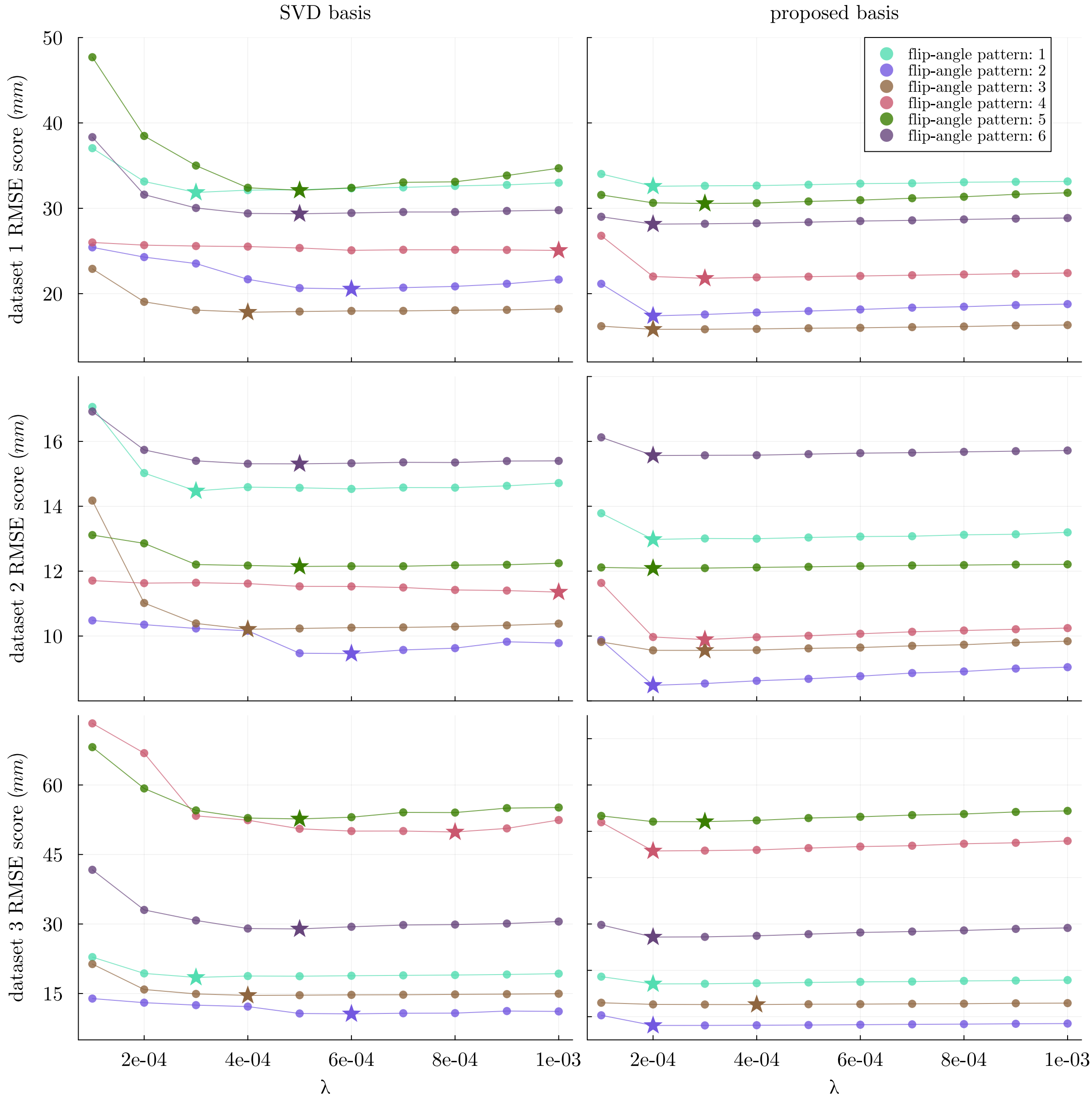

FIGURE S3 RMSE scores for simulated motion, calculated by comparing motion estimates to the ground truth. For both the SVD basis and the proposed contrast-optimized basis, we tested ten different regularization parameters λ to identify the optimal λ (star markers) for each flip-angle pattern. The proposed basis yields lower minimum RMSE scores for most flip-angle patterns across three motion patterns.

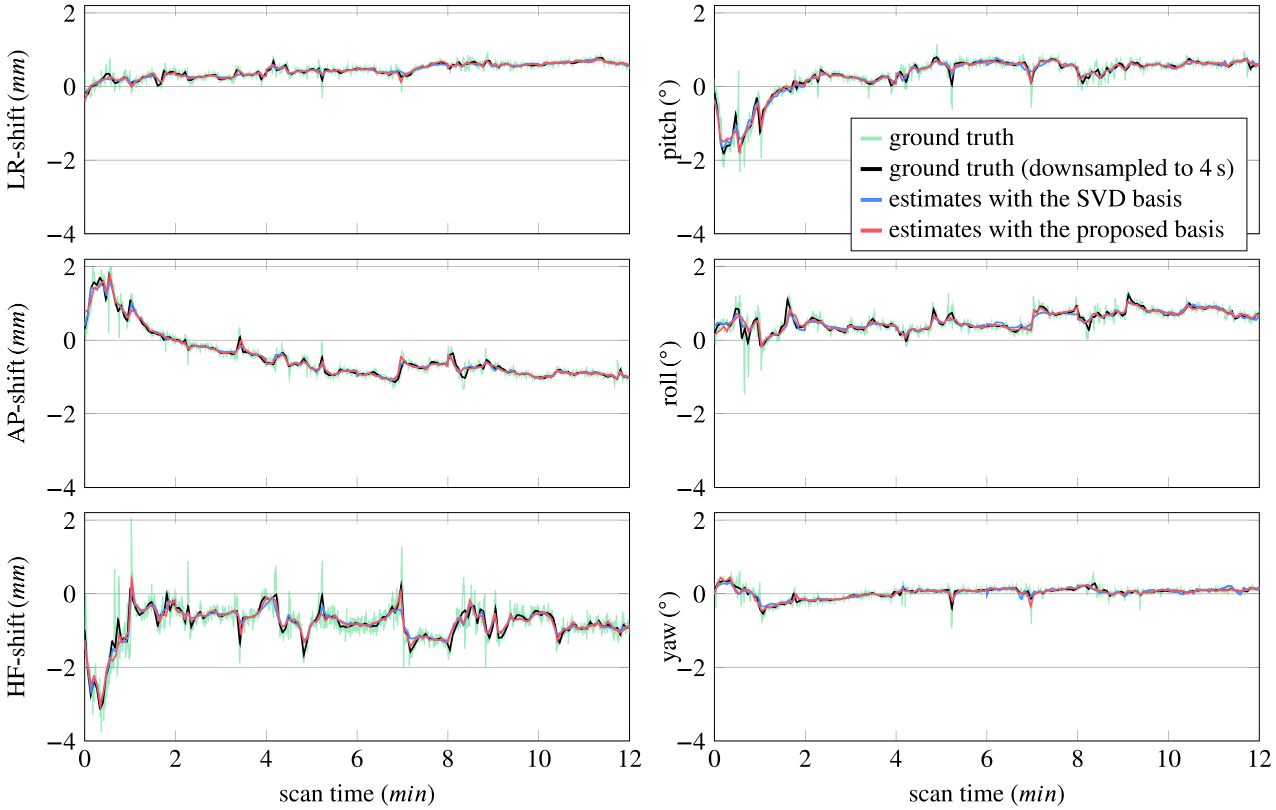

FIGURE S4 Estimates of moderate simulated motion (motion score of 1.77 mm). The ground truth was obtained from an online database. The RMSE score, which quantifies deviations of the motion estimates from the ground truth (Sec. 3.3.3) is 0.38 mm when using the SVD basis and 0.35 mm when using the proposed basis. Downsampling the ground-truth motion to 4 s windows provides a lower bound of 0.3 mm for the RMSE score, given the low temporal resolution.

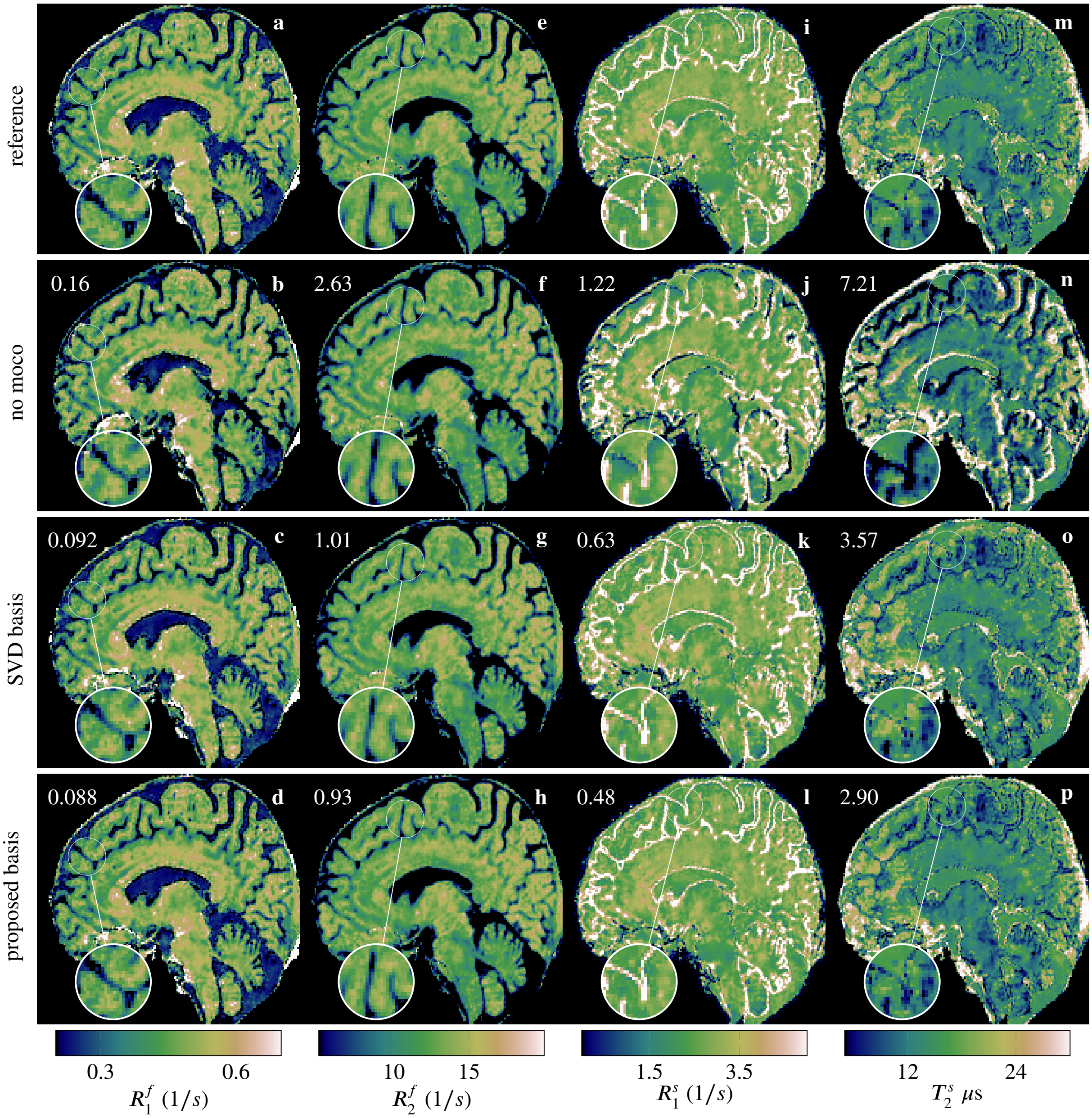

FIGURE S5 Parameter maps in the presence of moderate simulated motion, complementing the m_0^s and R_x maps in Fig. 3 of the main manuscript. The underlying data was corrupted with the pattern shown in Fig. 2 and Supporting Fig. S4. The RMSE for each parameter map, compared to the reference (no motion added), is shown in the top left of the respective map. The RMSE was calculated for the entire 3D brain volume, defined by the brain mask.

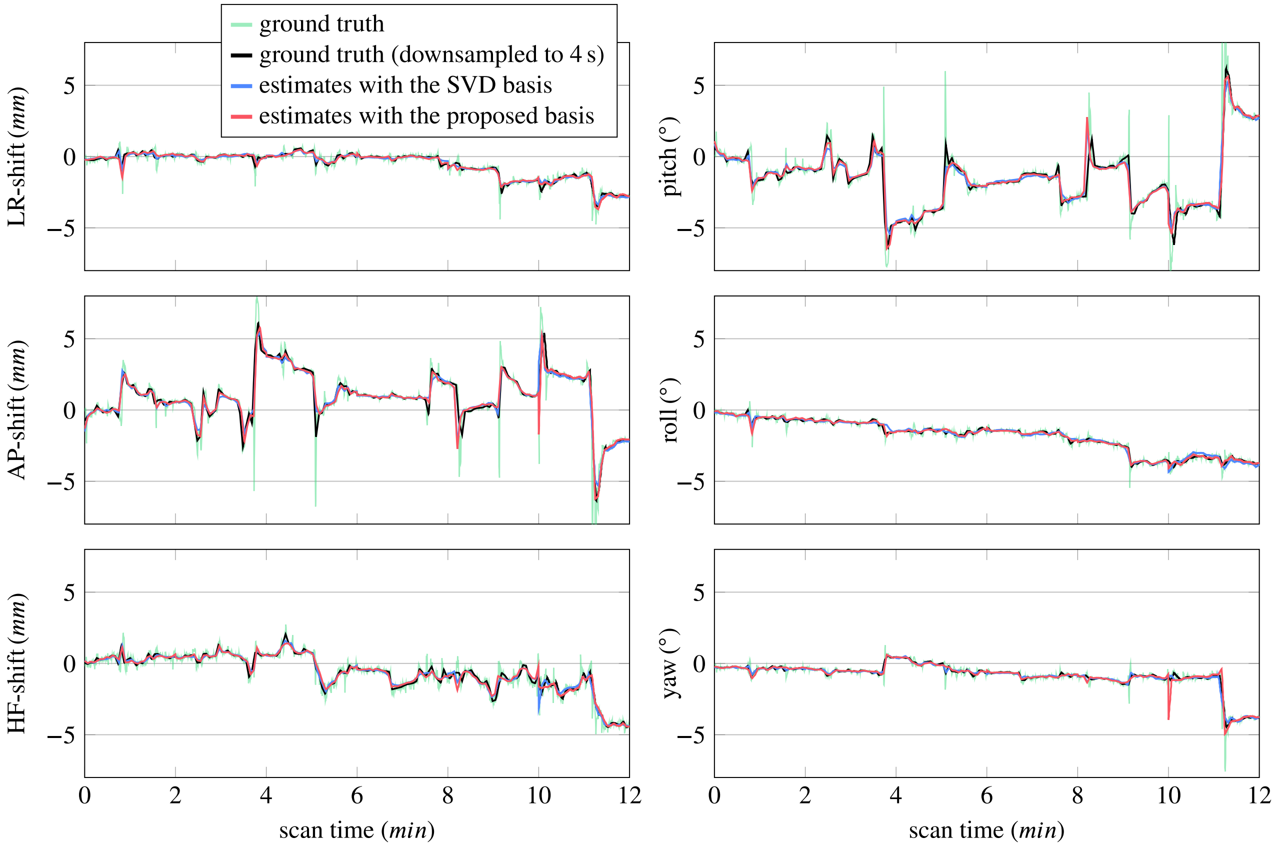

FIGURE S6 Estimates of strong simulated motion (motion score of 6.39 mm). The ground truth was obtained from an online database. The RMSE score, which quantifies deviations of the motion estimates from the ground truth (Sec. 3.3.3) is 1.33 mm when using the SVD basis and 1.26 mm when using the proposed basis. Downsampling the ground-truth motion to 4 s windows provides a lower bound of 1 mm for the RMSE score, given the low temporal resolution.

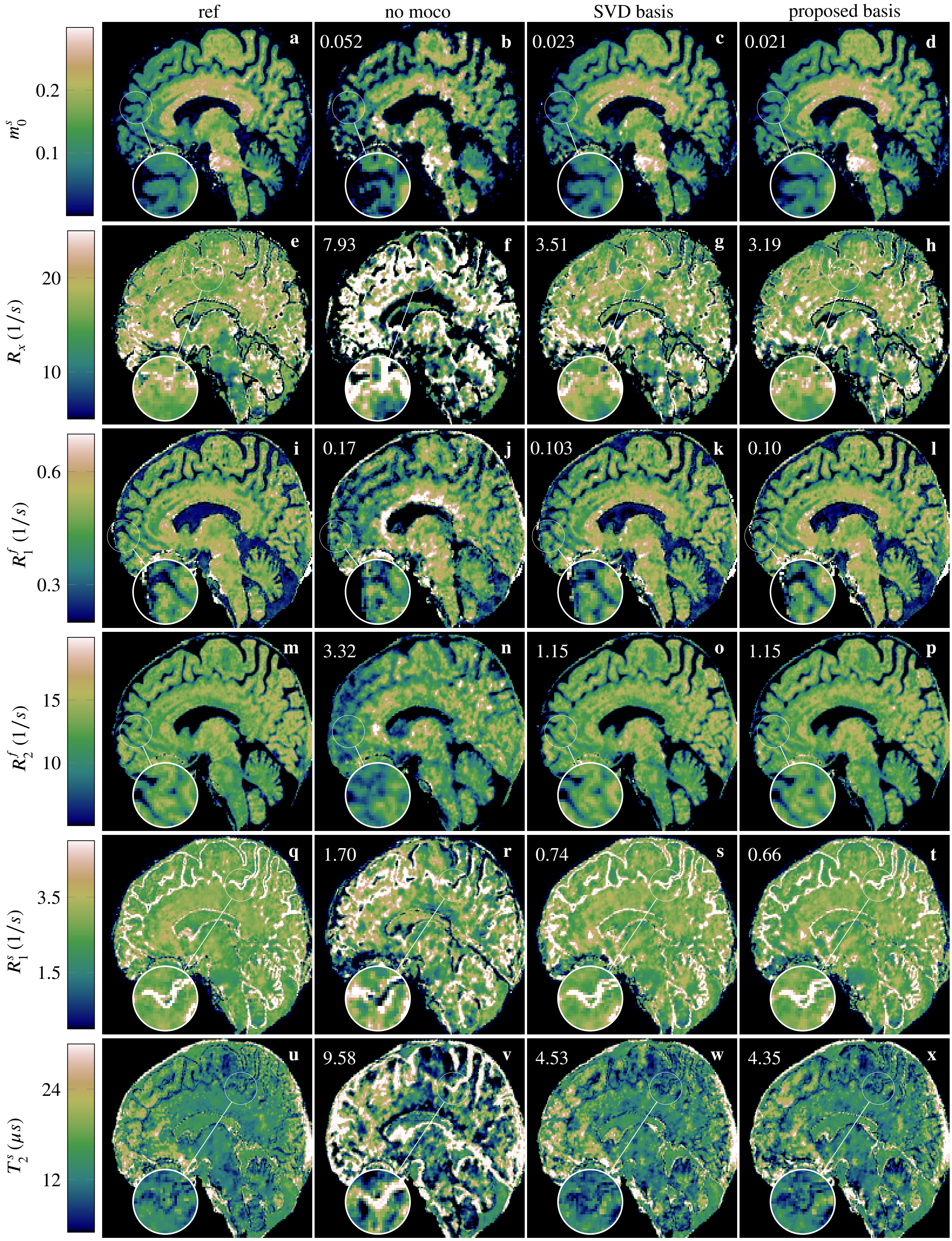

FIGURE S7 Parameter maps in the presence of strong simulated motion. The underlying data was corrupted with the pattern shown in Fig. S6. The RMSE for each parameter map, compared to the reference (no motion added), is shown in the top left of the respective map. The RMSE was calculated for the entire 3D brain volume, defined by the brain mask.

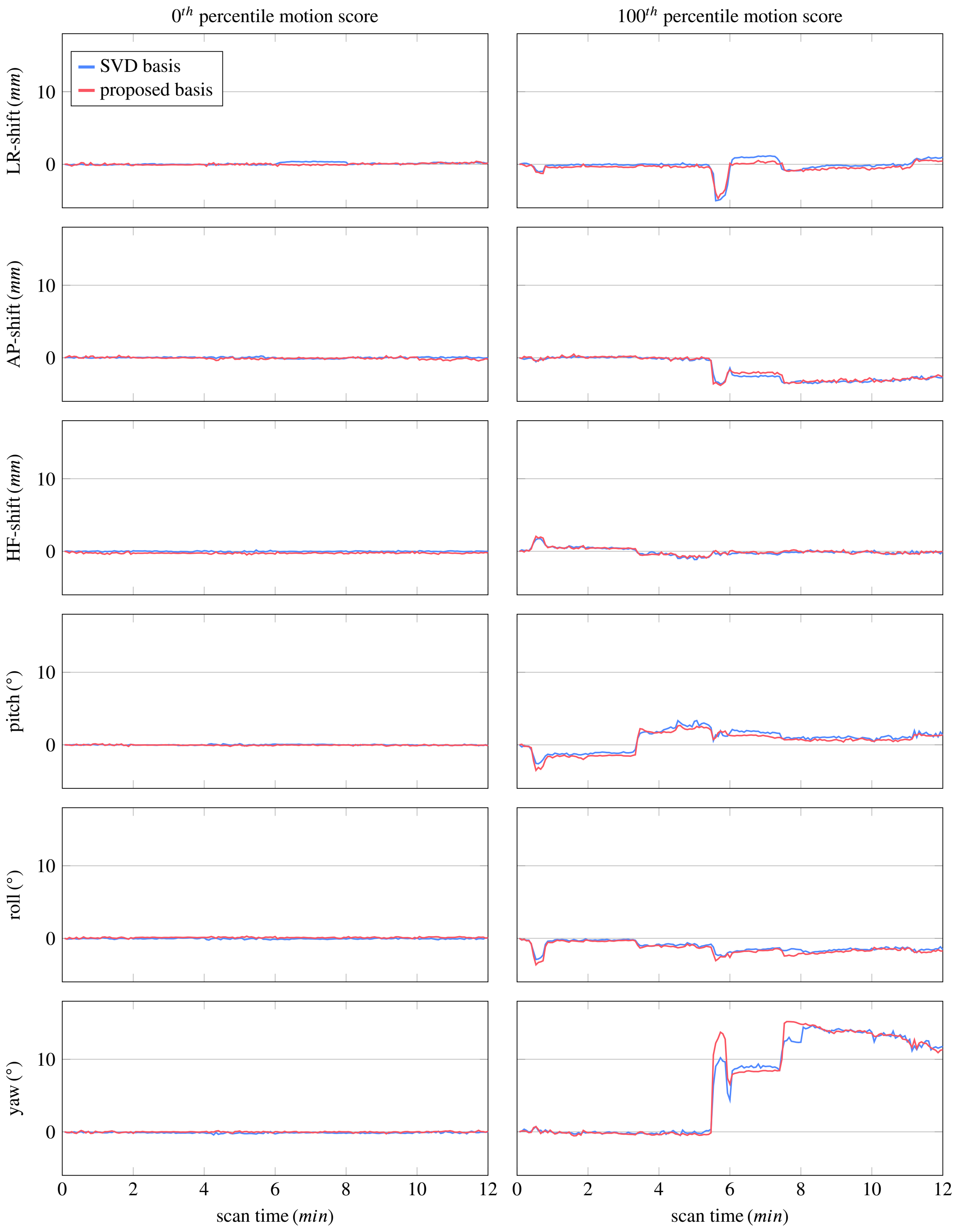

FIGURE S8 Estimates of inherent motion using the SVD basis [11] and the proposed contrast-optimized basis. We compare here a case with minimal (0th percentile motion score) and very strong (100th percentile motion score) unintended motion. The corresponding parametric maps can be found in Fig. 5 and Supporting Figs. S9 and S10.

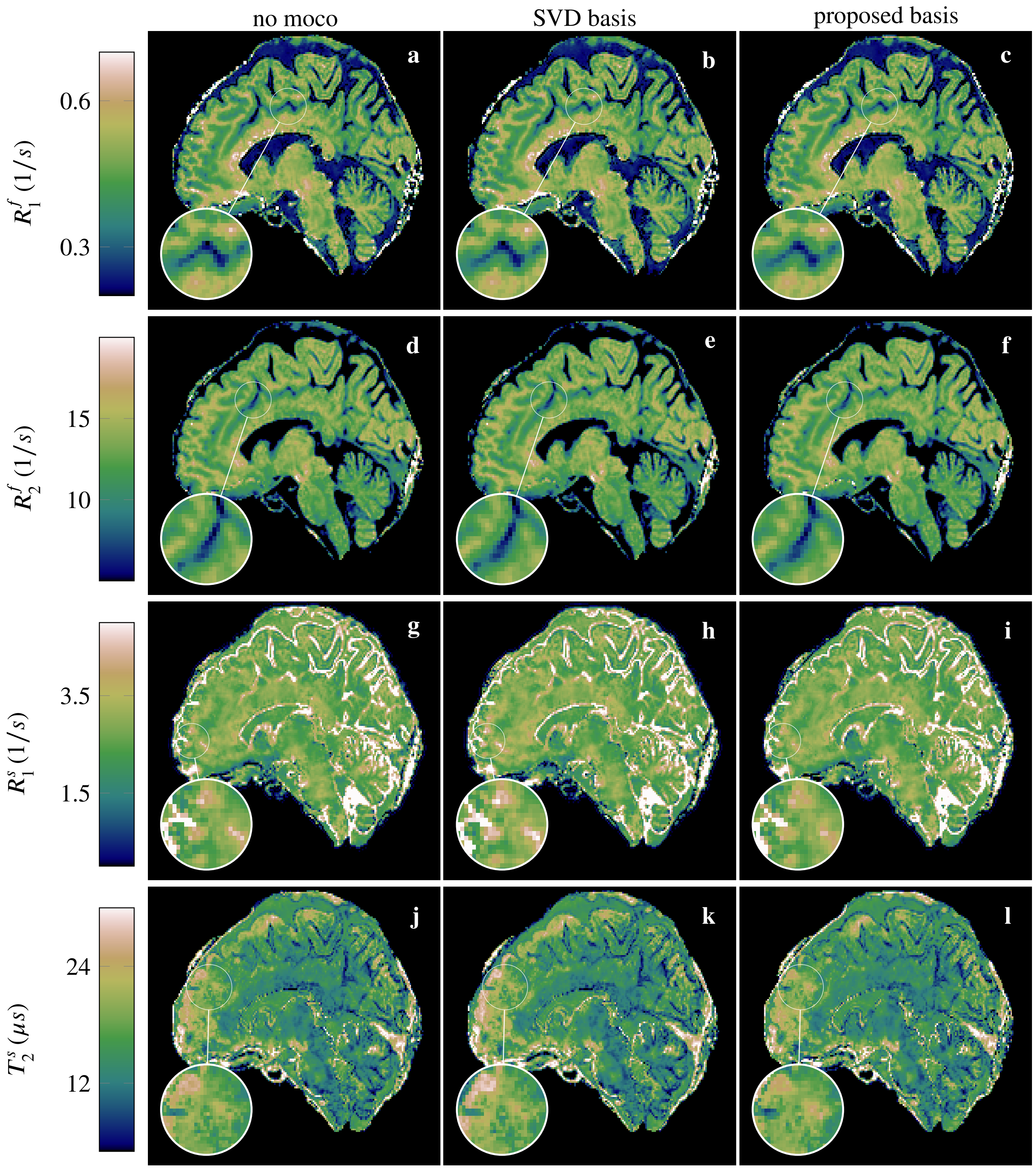

FIGURE S9 Parameter maps in the presence of minimal inherent motion (0th percentile motion score), complementing the m_0^s and R_x maps in Fig. 5 of the main manuscript. In this case, all three reconstructions have virtually identical results.

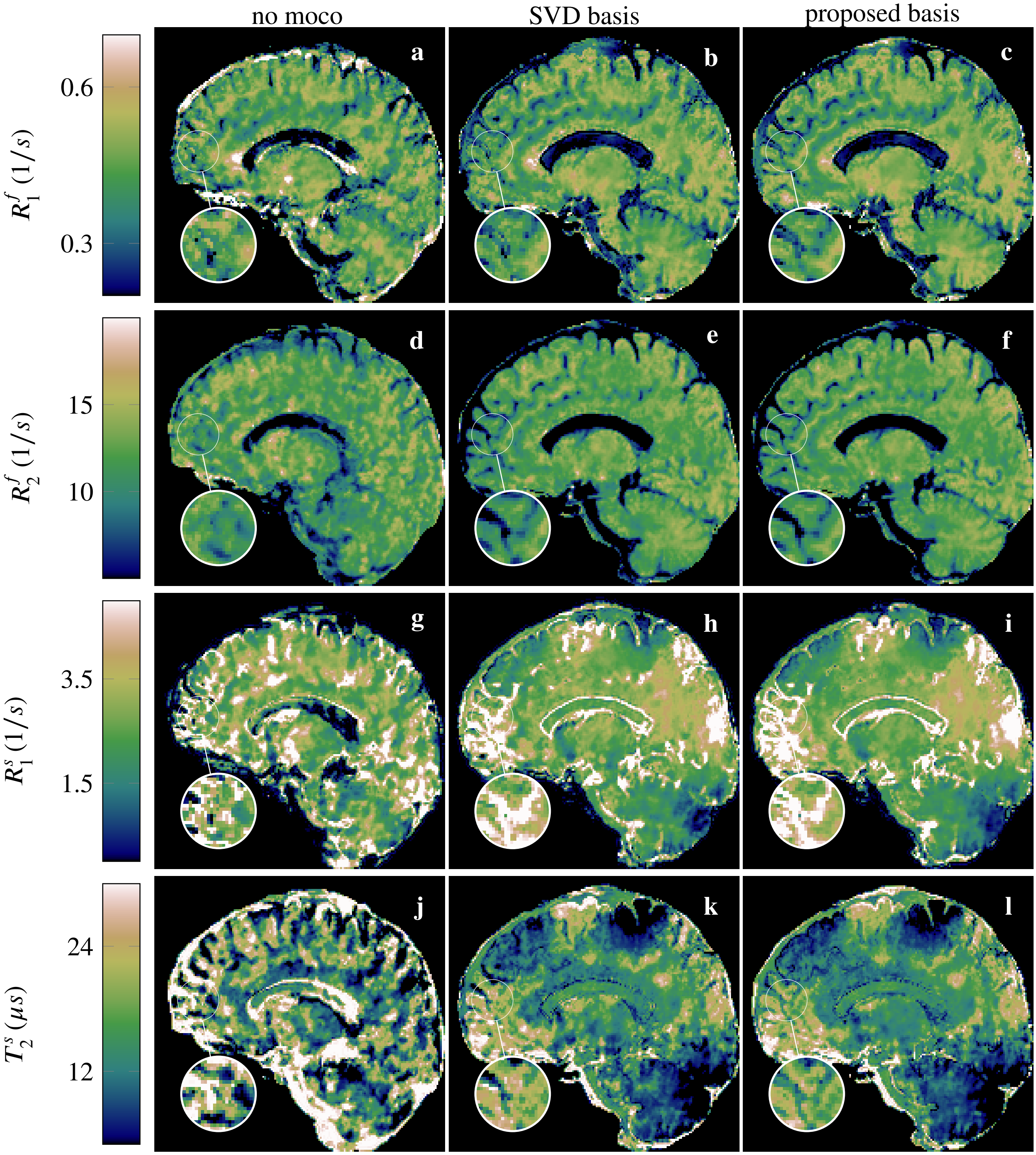

FIGURE S10 Parameter maps in the presence of very strong inherent motion (100th percentile motion score), complementing the m_0^s and R_x maps in Fig. 5 of the main manuscript.

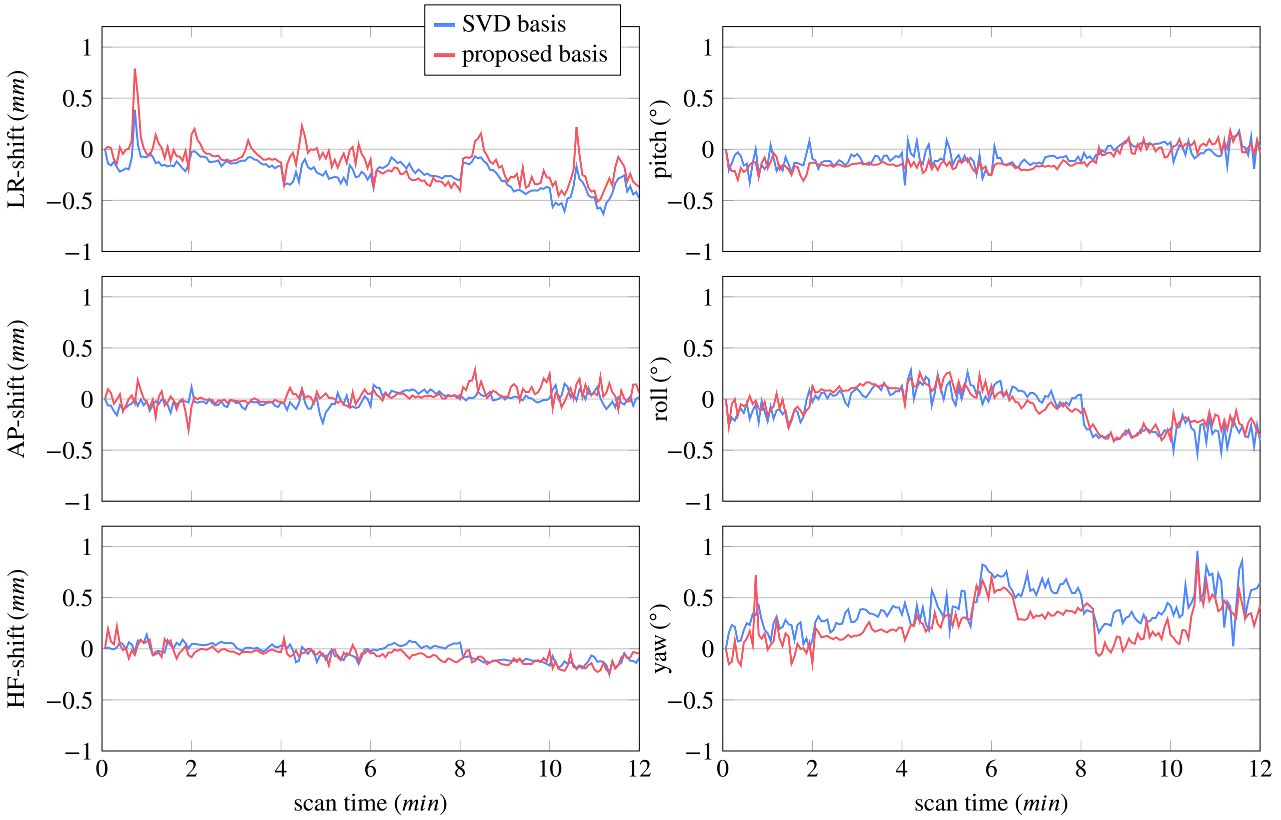

FIGURE S11 Estimates of inherent motion using the SVD basis [11] and the proposed contrast-optimized basis. We show here a case with a small amount of unintended motion (motion score 0.65 mm, 25th percentile; no data removed). The corresponding parametric maps can be found in the Supporting Fig. S12.

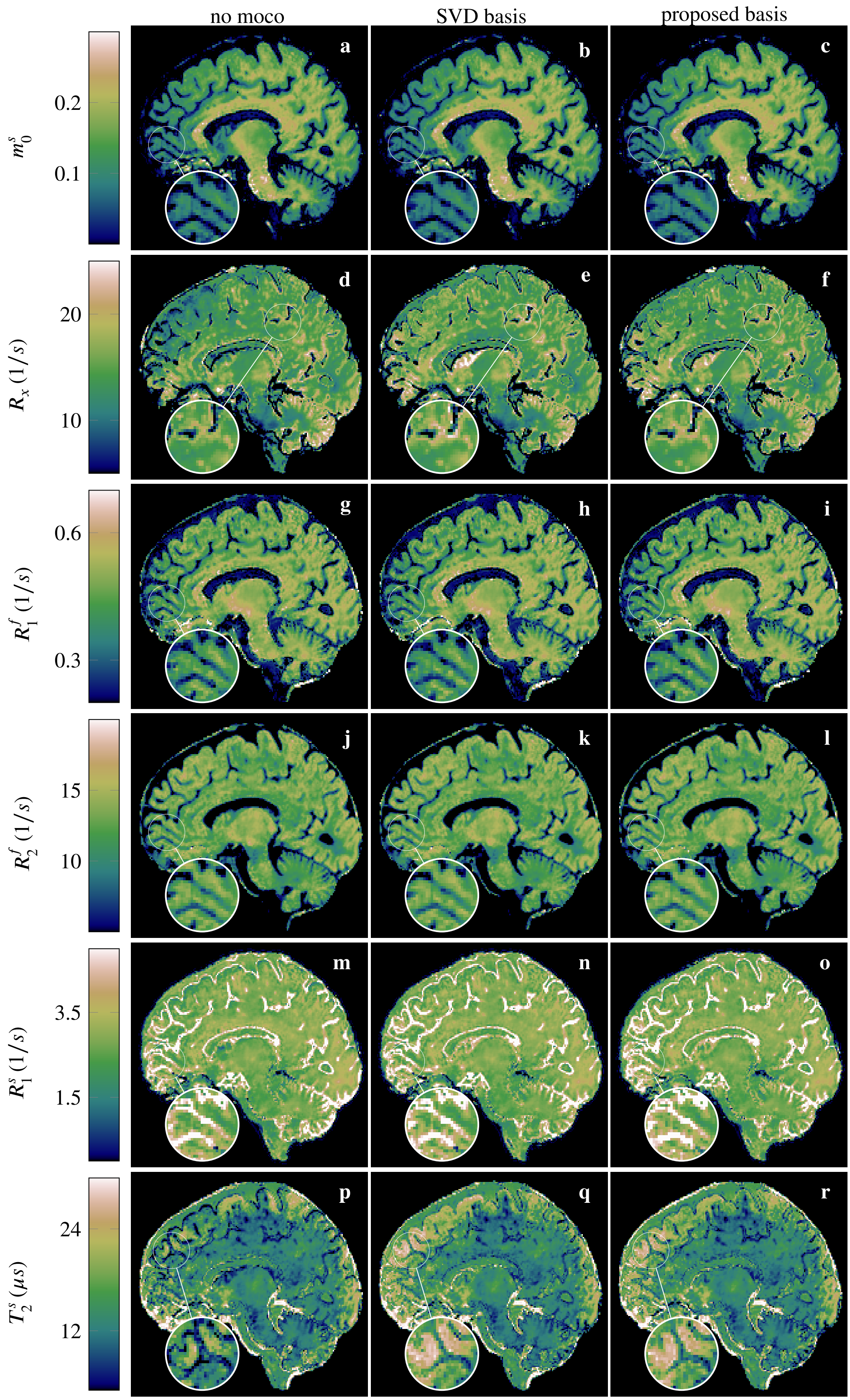

FIGURE S12 Parameter maps in the presence of small inherent motion (25th percentile motion score). The corresponding motion parameters can be found in the Supporting Fig. S11).

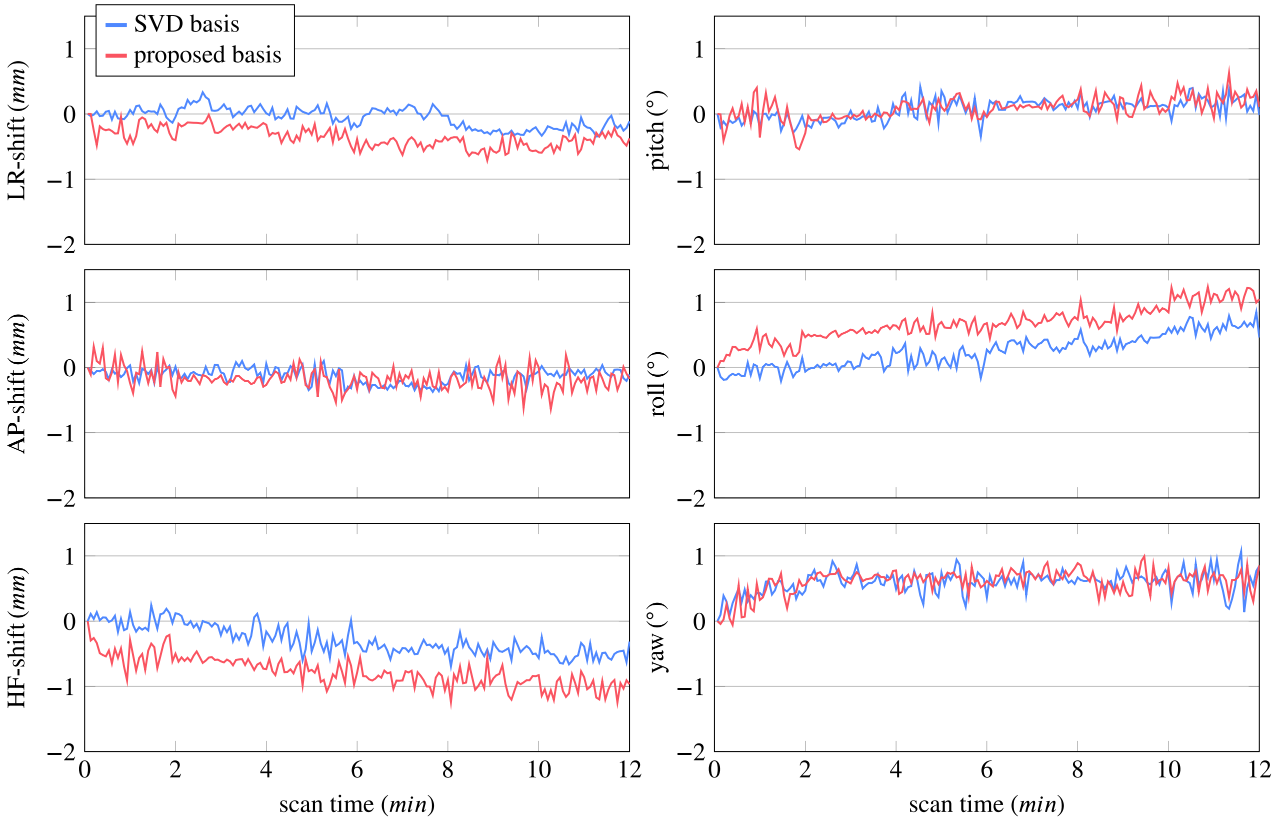

FIGURE S13 Estimates of inherent motion using the SVD basis [11] and the proposed contrast-optimized basis. We show here a case with an average amount of unintended motion (motion score 0.91 mm, 50th percentile; 1.11% of data removed). The corresponding parametric maps can be found in the Supporting Fig. S14.

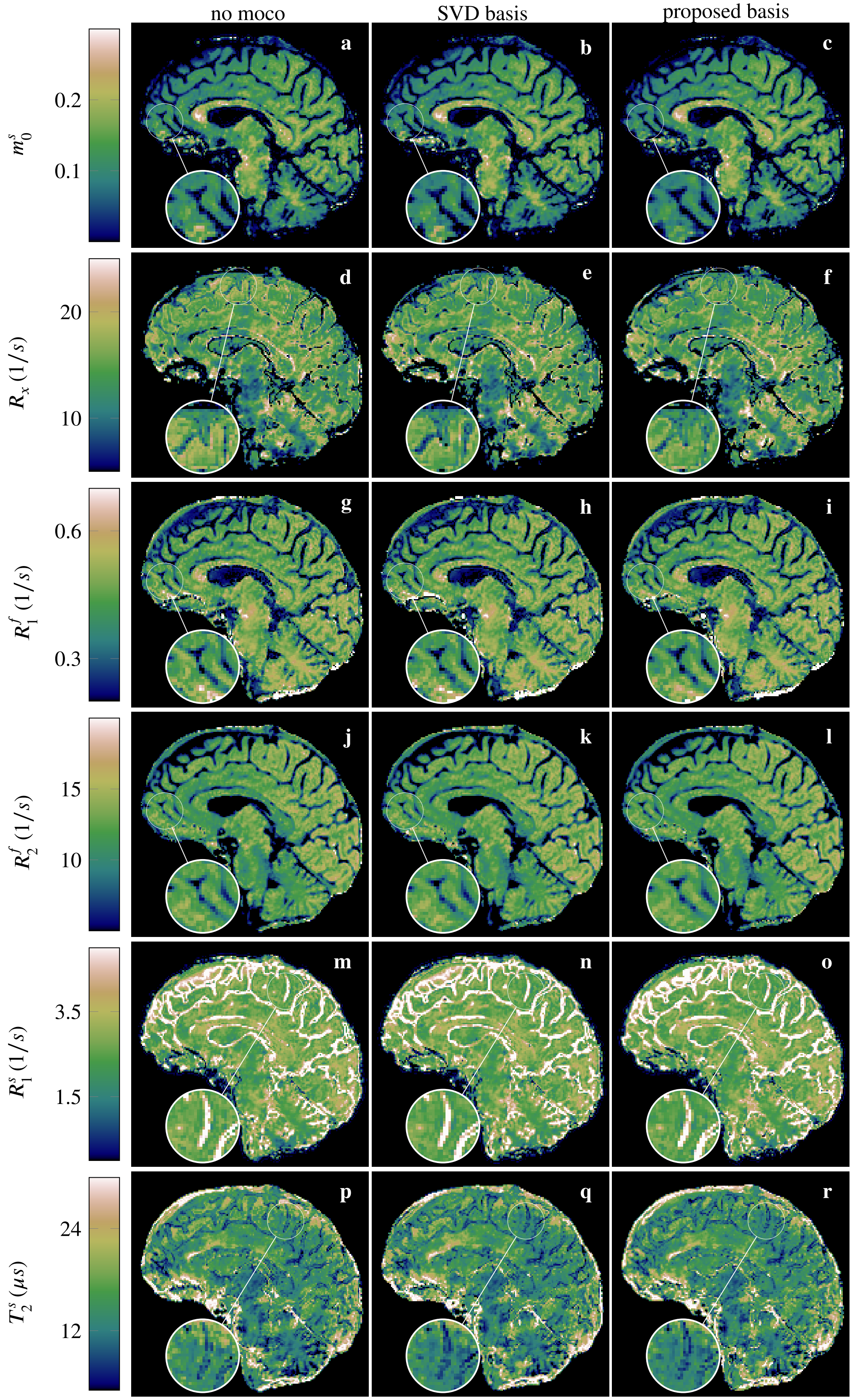

FIGURE S14 Parameter maps in the presence of average inherent motion (50th percentile motion score). The corresponding motion parameters can be found in the Supporting Fig. S13).

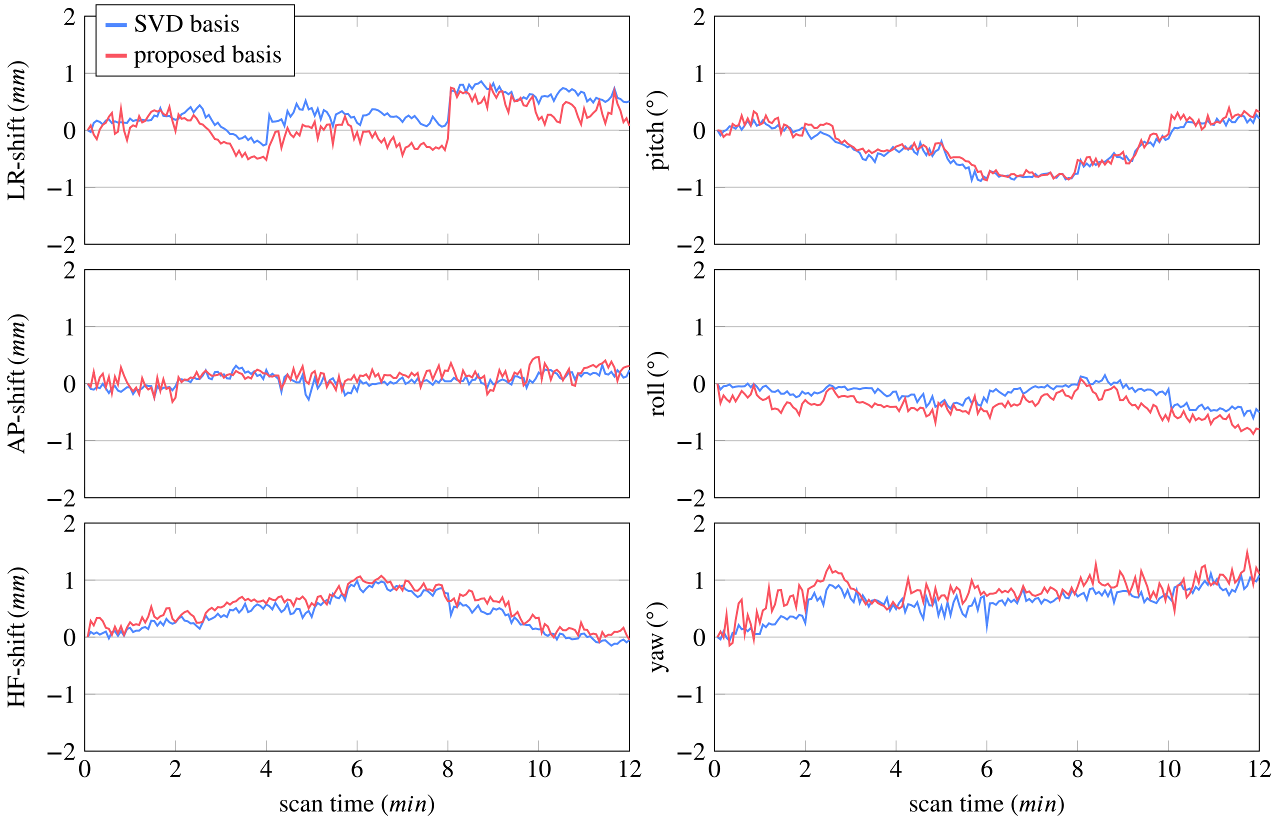

FIGURE S15 Estimates of inherent motion using the SVD basis [11] and the proposed contrast-optimized basis. We show here a case with a substantial amount of unintended motion (motion score 1.28 mm, 75th percentile; no data removed). The corresponding parametric maps can be found in the Supporting Fig. S16.

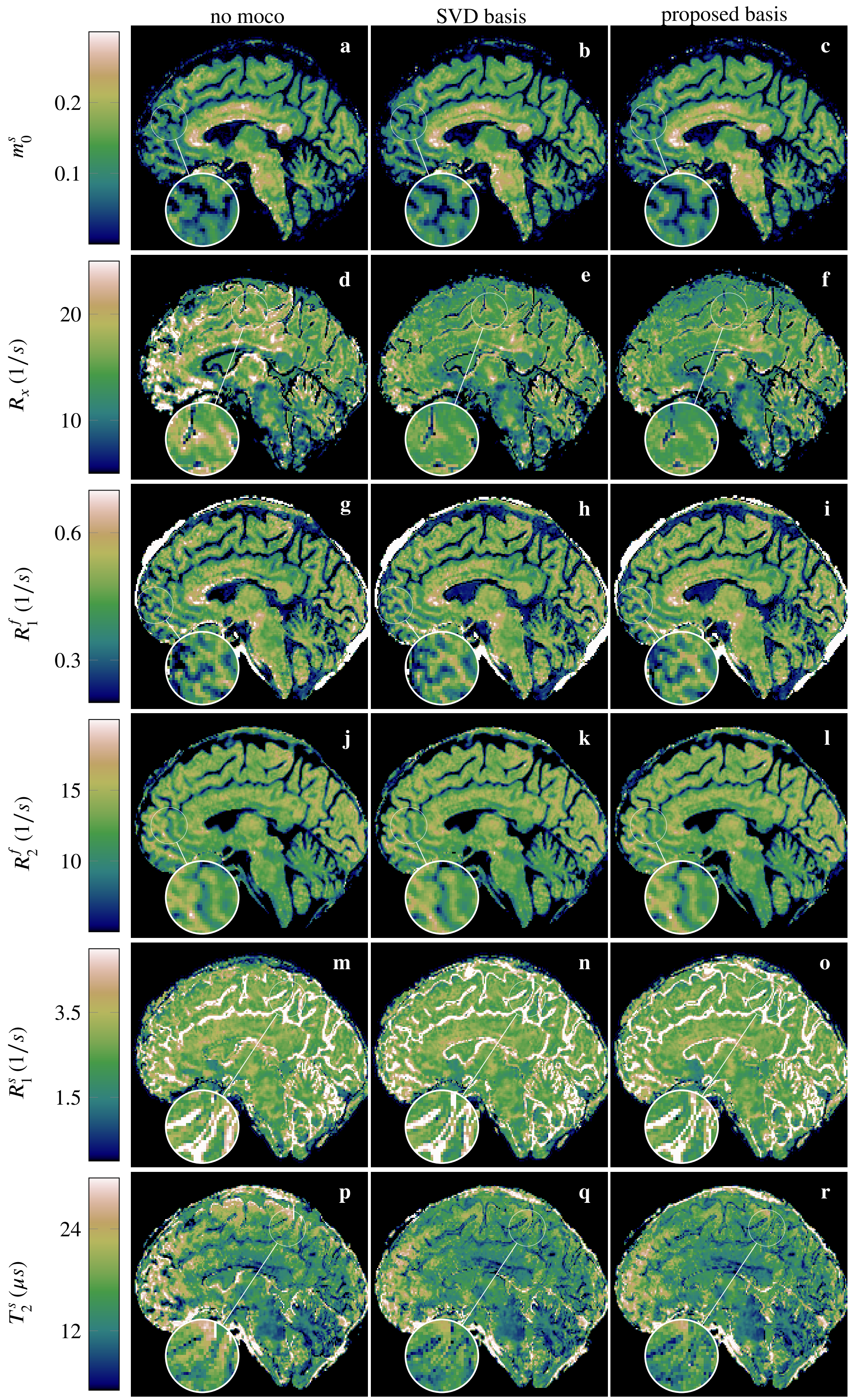

FIGURE S16 Parameter maps in the presence of substantial inherent motion (75th percentile motion score). The corresponding motion parameters can be found in the Supporting Fig. S15).

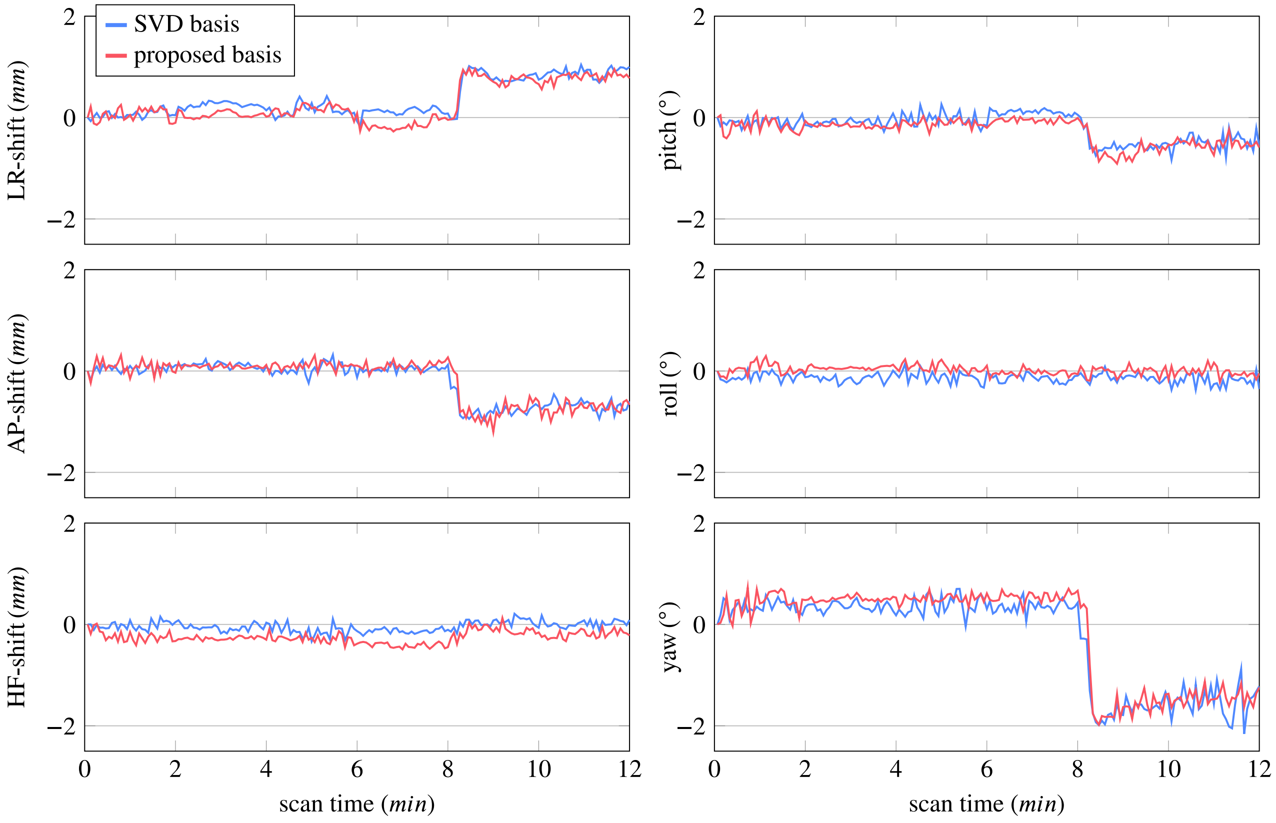

FIGURE S17 Estimates of inherent motion using the SVD basis [11] and the proposed contrast-optimized basis. We show here a case with a substantial amount of unintended motion (motion score 1.82 mm, 85th percentile; 1.11% of data removed). The corresponding parametric maps can be found in the Supporting Fig. S18.

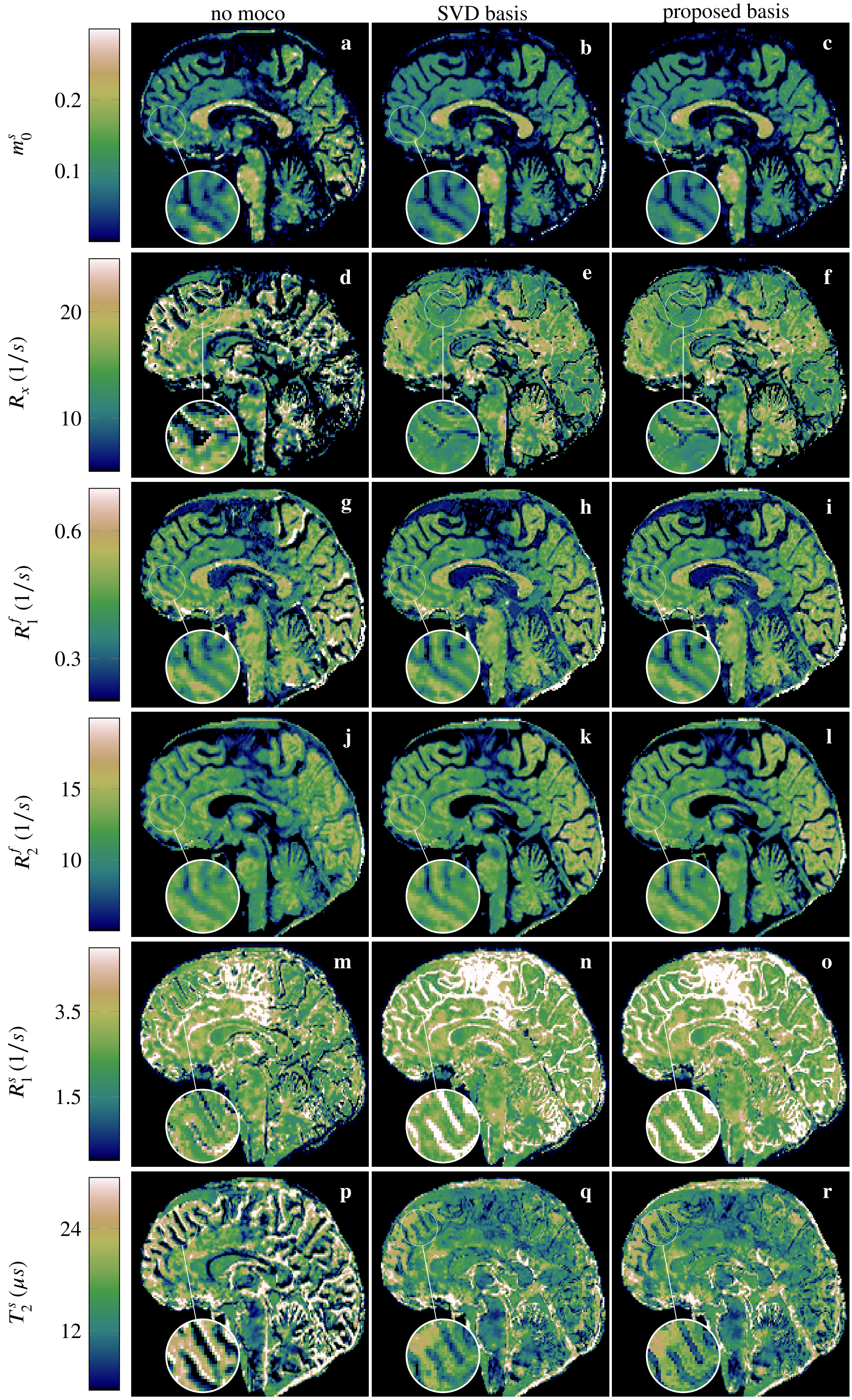

FIGURE S18 Parameter maps in the presence of substantial inherent motion (85th percentile motion score). The corresponding motion parameters can be found in the Supporting Fig. S17).

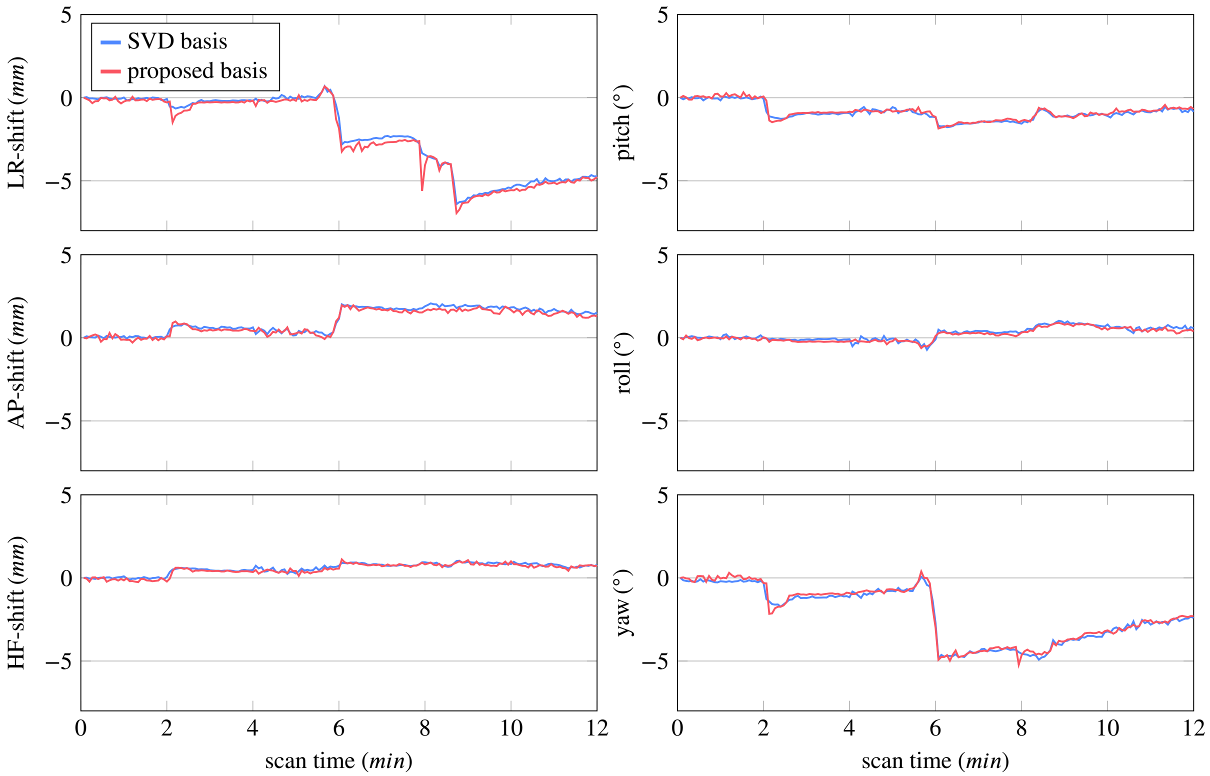

FIGURE S19 Estimates of inherent motion using the SVD basis [11] and the proposed contrast-optimized basis. We show here a case with an extreme amount of unintended motion (motion score 5.17 mm, 95th percentile; 6.11% of data removed). The corresponding parametric maps can be found in the Supporting Fig. S20.

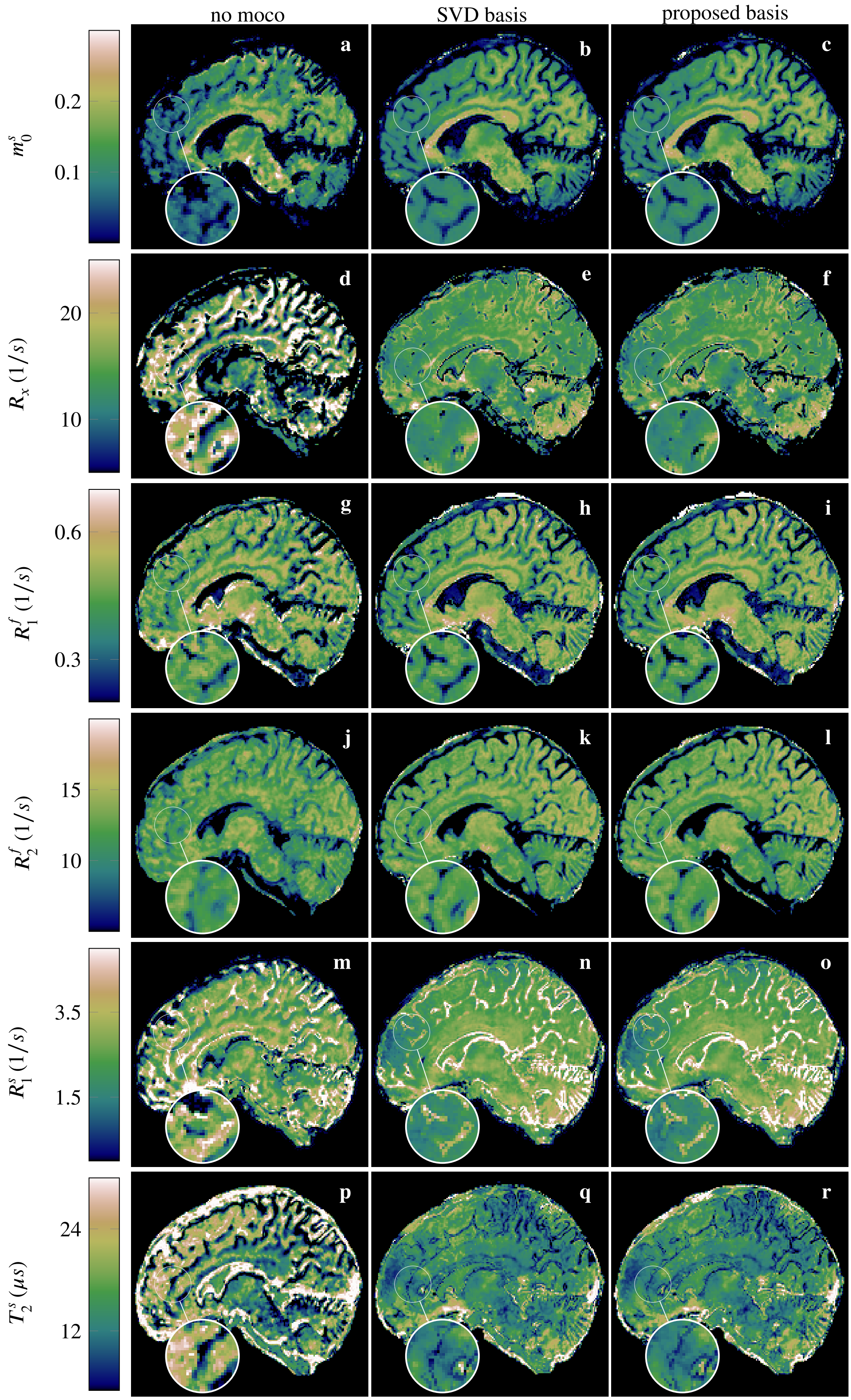

FIGURE S20 Parameter maps in the presence of extreme inherent motion (95th percentile motion score). The corresponding motion parameters can be found in the Supporting Fig. S19).

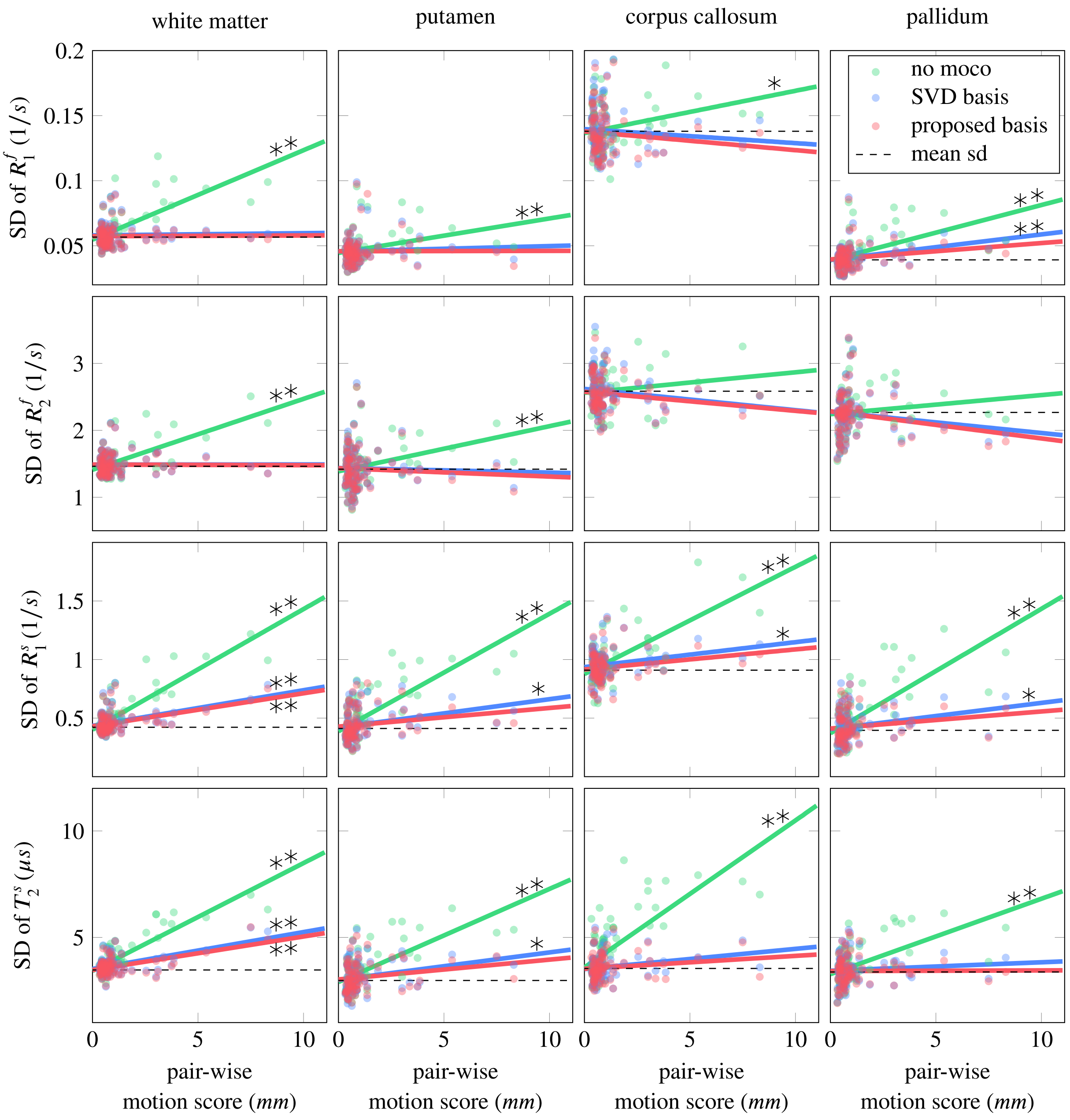

FIGURE S21 Analysis of noise-like artifacts across all 85 datasets, complementing the analysis of m_0^s and R_x in Fig. 6. Each dot represents the standard deviation of the qMT parameter for one participant. The standard deviation was calculated for each motion correction version methods. Linear regression was performed to analyze the increase in the standard deviation with increasing motion. Black stars denote slopes that differ significantly from zero (* for p-value < 0.05, ** for p-value < 0.01). The slopes of this linear regression analysis are further analyzed in Fig. 7.

The corresponding parametric maps can be found in the Supporting Fig. S16.

Figure S16: Parameter maps in the presence of substantial inherent motion (75th percentile motion score). The corresponding motion parameters can be found in the Supporting Fig. S15).

Figure S17: Estimates of inherent motion using the SVD basis [14] and the proposed contrast-optimized basis. We show here a case with a substantial amount of unintended motion (motion score 1.82 mm, 85th percentile; 1.11% of data removed). The corresponding parametric maps can be found in the Supporting Fig. S18.

Figure S18: Parameter maps in the presence of substantial inherent motion (85th percentile motion score). The corresponding motion parameters can be found in the Supporting Fig. S17).

Figure S19: Estimates of inherent motion using the SVD basis [14] and the proposed contrast-optimized basis. We show here a case with an extreme amount of unintended motion (motion score 5.17 mm, 95th percentile; 6.11% of data removed). The corresponding parametric maps can be found in the Supporting Fig. S20.

Figure S20: Parameter maps in the presence of extreme inherent motion (95th percentile motion score). The corresponding motion parameters can be found in the Supporting Fig. S19).

Figure S21: Analysis of noise-like artifacts across all 85 datasets, complementing the analysis of m_0^s and R_x in Fig. 6. Each dot represents the standard deviation of the qMT parameter for one participant. The standard deviation was calculated for each motion correction version methods. Linear regression was performed to analyze the increase in the standard deviation with increasing motion. Black stars denote slopes that differ significantly from zero (* for p-value < 0.05, ** for p-value < 0.01). The slopes of this linear regression analysis are further analyzed in Fig. 7.

REFERENCES

1. Huber Peter J. Robust estimation of a location parameter. In: Springer 1992 (pp. 492–518).
2. Ashburner John, Barnes Gareth, Chen Chun-Chuan, et al. SPM12 manual. *Wellcome Trust Centre for Neuroimaging, London, UK.* 2014;2464(4).
3. Kelley William, Ngo Nathan, Dalca Adrian V, Fischl Bruce, Zöllei Lilla, Hoffmann Malte. *Boosting skull-stripping performance for pediatric brain images.* 2024.
4. Hoopes Andrew, Mora Jocelyn S, Dalca Adrian V, Fischl Bruce, Hoffmann Malte. SynthStrip: Skull-stripping for any brain image. *NeuroImage.* 2022;260:119474.
5. Lustig Michael, Donoho David, Pauly John M. Sparse MRI: The application of compressed sensing for rapid MR imaging. *Magnetic Resonance in Medicine: An Official Journal of the International Society for Magnetic Resonance in Medicine.* 2007;58(6):1182–1195.
6. Zhang Tao, Pauly John M, Levesque Ives R. Accelerating parameter mapping with a locally low rank constraint. *Magnetic resonance in medicine.* 2015;73(2):655–661.
7. Trzasko Joshua, Manduca Armando, Borisch Eric. Local versus global low-rank promotion in dynamic MRI series reconstruction. *Proc. Int. Symp. Magn. Reson. Med.* 2011;19:4371.
8. Jang Uijeong, Gupta Shuvomoy Das, Ryu Ernest K. Computer-Assisted Design of Accelerated Composite Optimization Methods: OptISTA. *arXiv preprint arXiv:2305.15704.* 2023;.
9. Reuter Martin, Rosas H Diana, Fischl Bruce. Highly accurate inverse consistent registration: a robust approach. *Neuroimage.* 2010;53(4):1181–1196.
10. Tisdall M Dylan, Hess Aaron T, Reuter Martin, Meintjes Ernesta M, Fischl Bruce, Kouwe André JW. Volumetric navigators for prospective motion correction and selective reacquisition in neuroanatomical MRI. *Magnetic resonance in medicine.* 2012;68(2):389–399.
11. Kurzawski Jan W, Cencini Matteo, Peretti Luca, et al. Retrospective rigid motion correction of three-dimensional magnetic resonance fingerprinting of the human brain. *Magnetic Resonance in Medicine.* 2020;84(5):2606–2615.
