# Supplementary material for: Contrast-Optimized Basis Functions for Self-Navigated Motion Correction in Quantitative MRI
Source: ArXiv. 2024 Dec 27:arXiv:2412.19552v1. Preprint. [Version 1] (PMC11703326)
Supplement: Supplementary file 1 [file NIHPP2412.19552v1-supplement-1.pdf]

SUPPORTING INFORMATION

S1 CONSENSUS-BASED MOTION ESTIMATES

The motion estimates relative to each sequence were repeatedly estimated, where each volume served as a reference to co-register all the remaining volumes (29) per each of the six flip angle patterns. This iterative approach was preferred over selecting a single reference per each sequence, to mitigate the risk of the chosen volume being affected by motion artifacts.

A weighted consensus estimate was then computed to minimize the influence of outliers. For a set of motion parameters $\{p_i\}$, the consensus estimate p_{cons} is iteratively updated as:

$$p_{\text{cons}}^{(k+1)} = \frac{\sum_i w_i^{(k)} p_i}{\sum_i w_i^{(k)}},$$

with k being the number of iterations, and the weights w_i are inversely proportional to the residual distances, $r_i = \|p_i - p_{\text{cons}}^{(k)}\|$, given by:

$$w_i^{(k)} = \frac{1}{1 + r_i}.$$

The iterative process continues until the changes in p_{cons} fall below a tolerance threshold. The so-derived affine matrices are then used for the k-space-based motion correction.

Additionally, to further improve the accuracy of our motion estimates, we co-registered high-resolution volumes derived from each of the six flip angle patterns. To this extent, we combined all the data from each flip angle pattern (2 min of acquisition), and reconstructed it in the subspace of the proposed contrast-optimized basis using the OptISTA algorithm[25] and locally low-rank regularization[29–31] to reduce residual noise on the final image. The resulting 1 mm isotropic volumes (6 in total, one per each sequence) were then co-registered using *mri_robust_register* from *Freesurfer*,[32] with the second sequence volume as reference. The motion estimates were then applied to the individual 4 s blocks of each flip angle pattern.

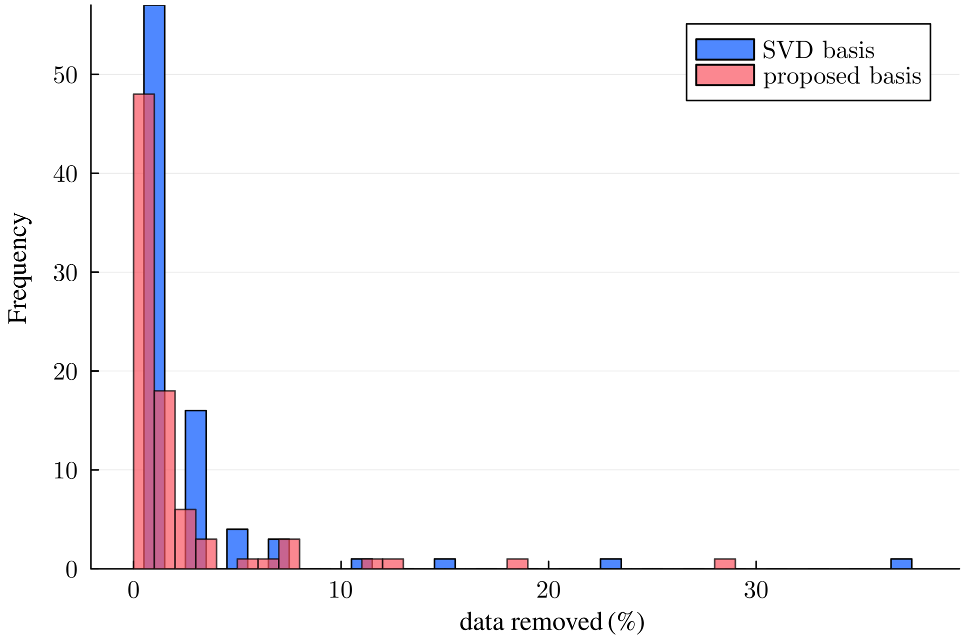

FIGURE S1 Percentage of data removal for all the datasets estimated using a motion score[21] on the motion estimates derived using the SVD basis[8] and the proposed contrast-optimized basis.

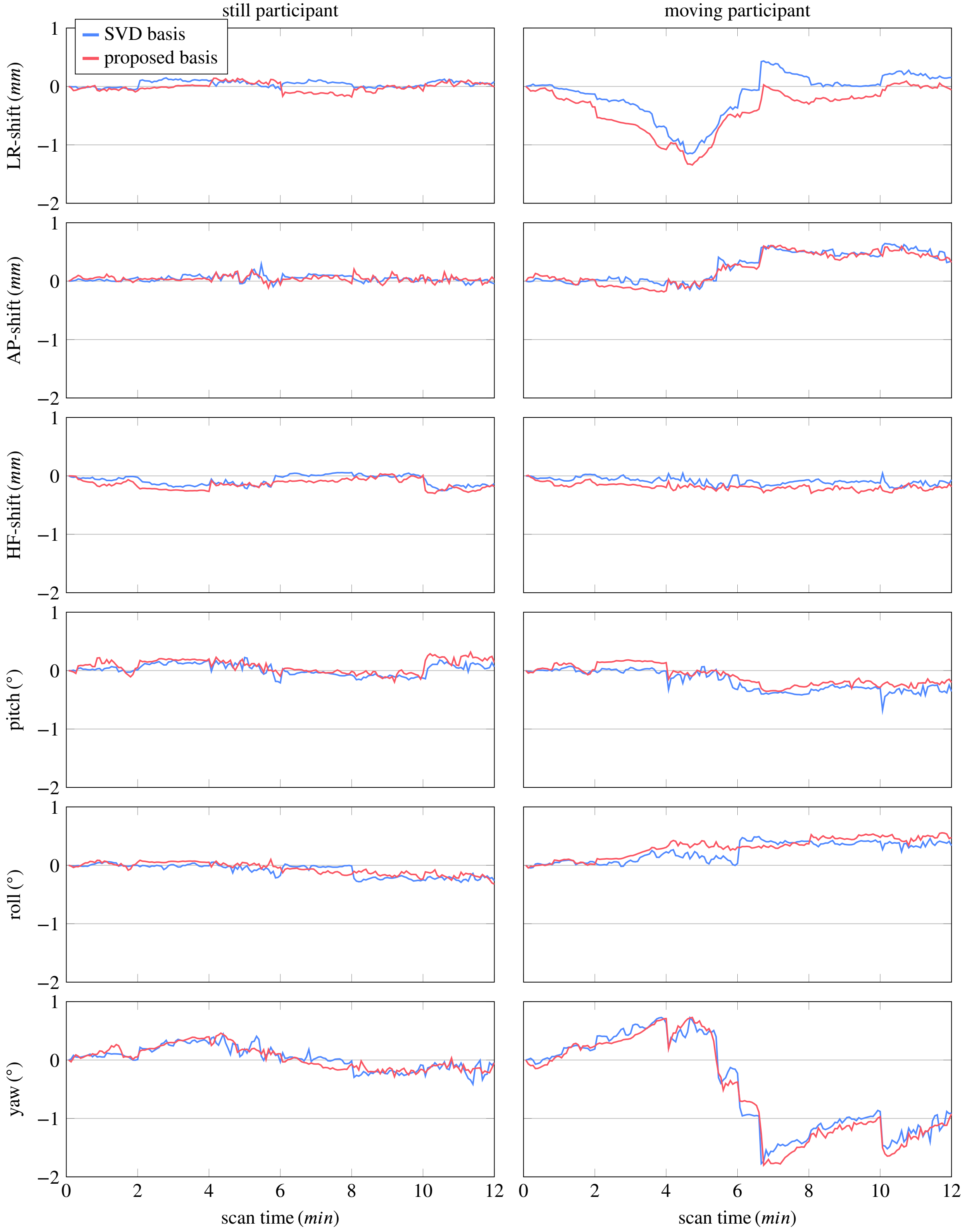

FIGURE S2 Motion parameters estimated using the SVD basis [8] and the proposed contrast-optimized basis in case of no voluntary motion ("still" participant) and in presence of voluntary motion ("moving" participant). The former led to no apparent degradation of the parametric maps, while the latter produced motion artifacts which are mitigated by the motion correction methods (Fig. 3 and Supporting Fig. S4).

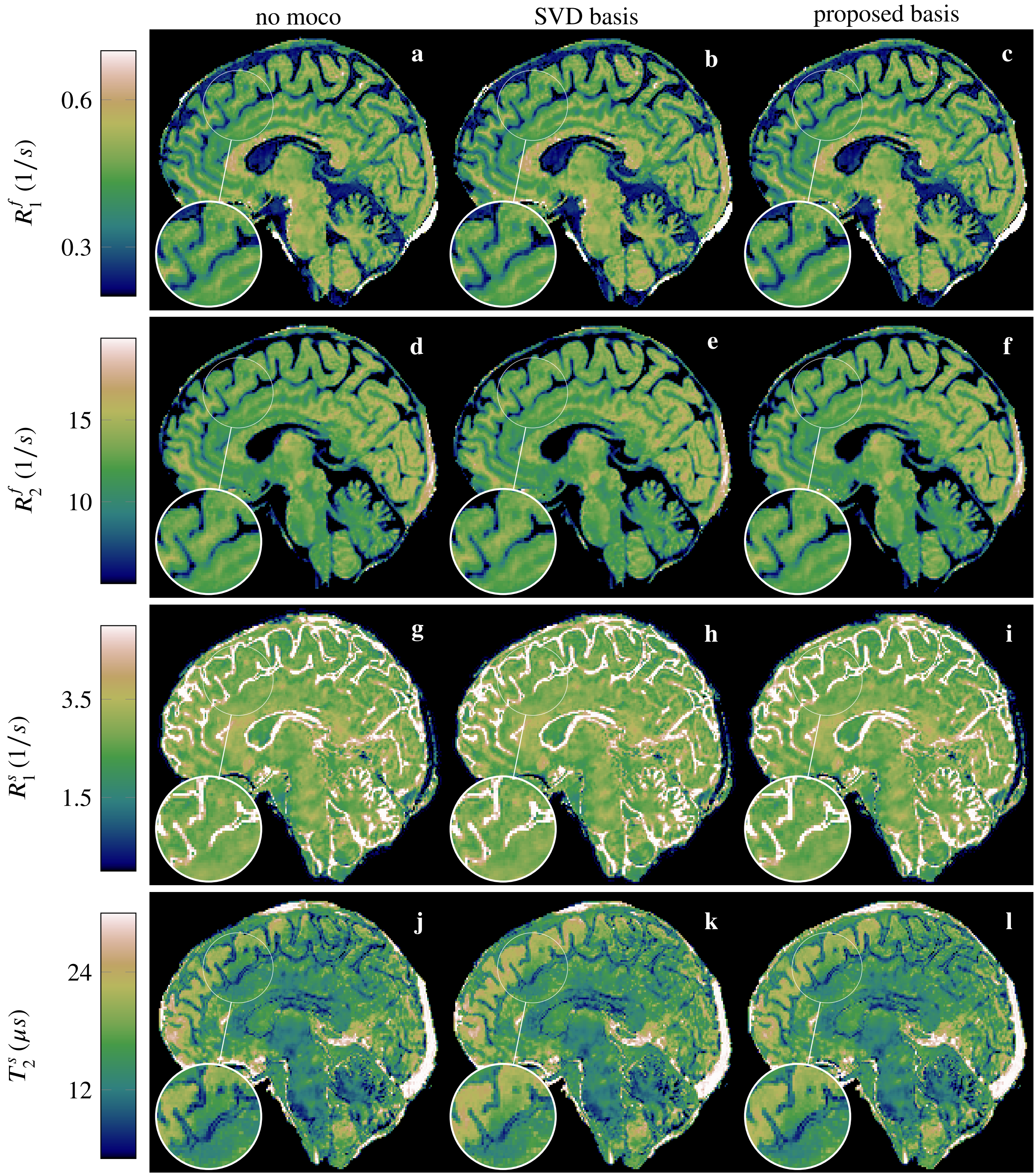

FIGURE S3 Sagittal view of the remaining qMT parameters maps (R_1^f, R_2^f, R_1^s, and T_2^s) of a scan with virtually no motion ("still" participant), with the three reconstruction methods showing visually identical results.

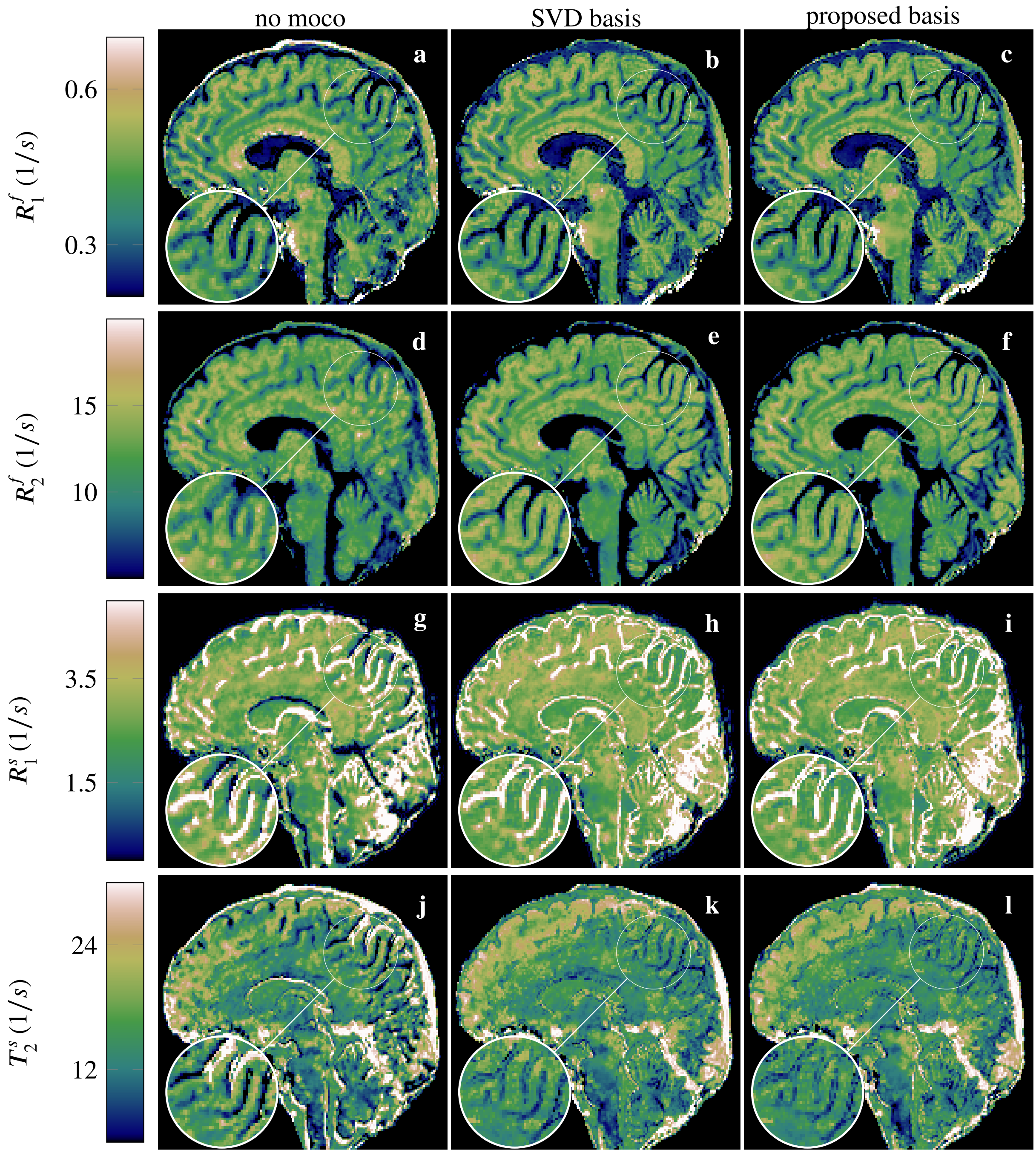

FIGURE S4 Sagittal view of the remaining qMT parameters maps (R_1^f, R_2^f, R_1^s, and T_2^s) of a representative acquisition, where the subject exhibited a motion score at the 87th percentile ("moving" participant), with the three reconstruction methods showing visually identical results.

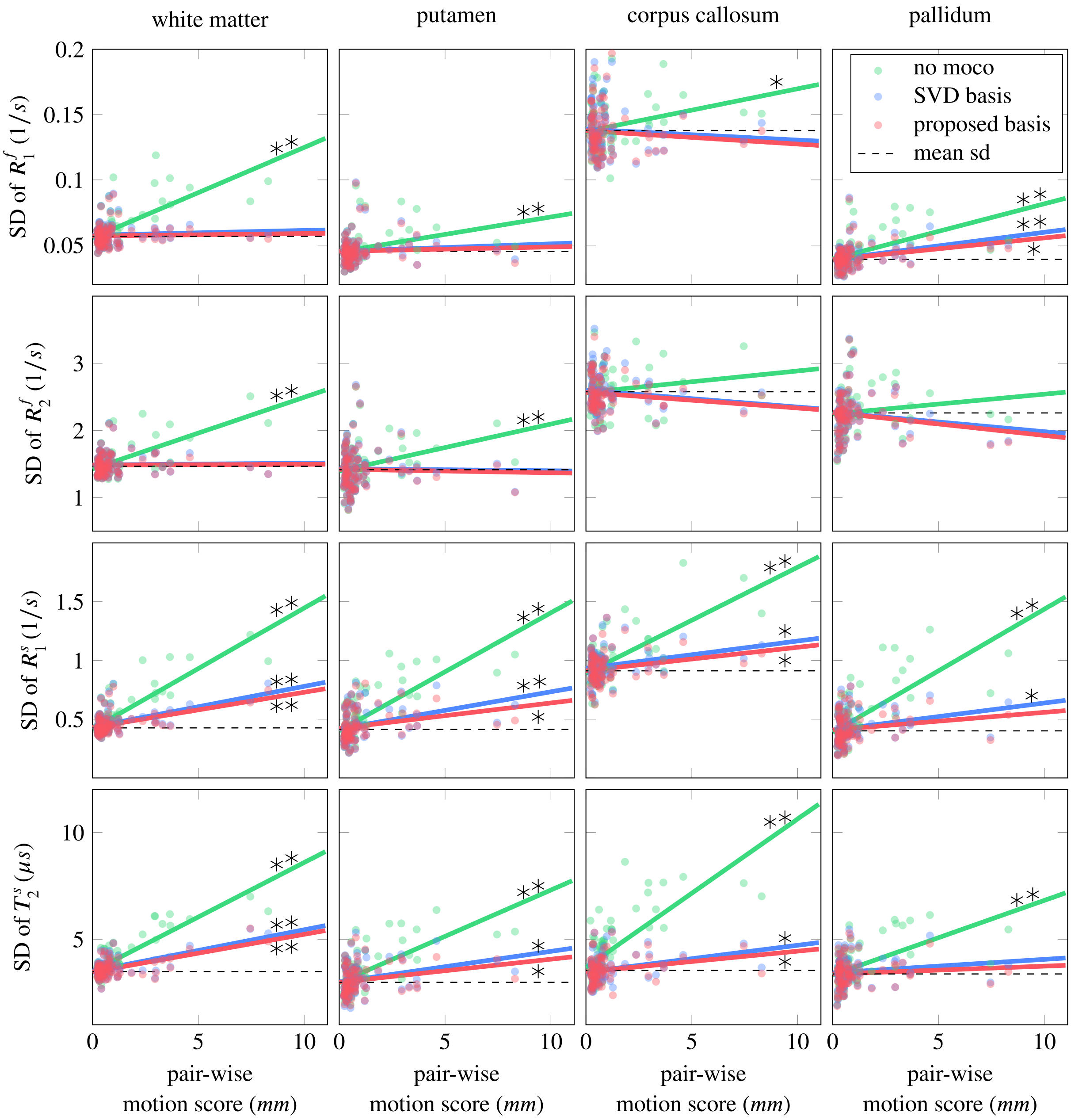

FIGURE S5 Each dot represents the standard deviation of the qMT parameter for one participant. The mean standard deviation was calculated across the three reconstruction methods. Linear regression was performed to analyze the increase of the standard deviation with increasing motion. Black stars denote slopes that differ significantly from zero (* for p-value < 0.05, ** for p-value < 0.01). The motion correction using the estimates derived using the SVD basis provide a considerable reduction in the slopes of the linear regression curves, with most being non-significantly different from zero, which indicates effective motion artifact compensation. The slopes of this linear regression analysis are further analyzed in Fig. 4.

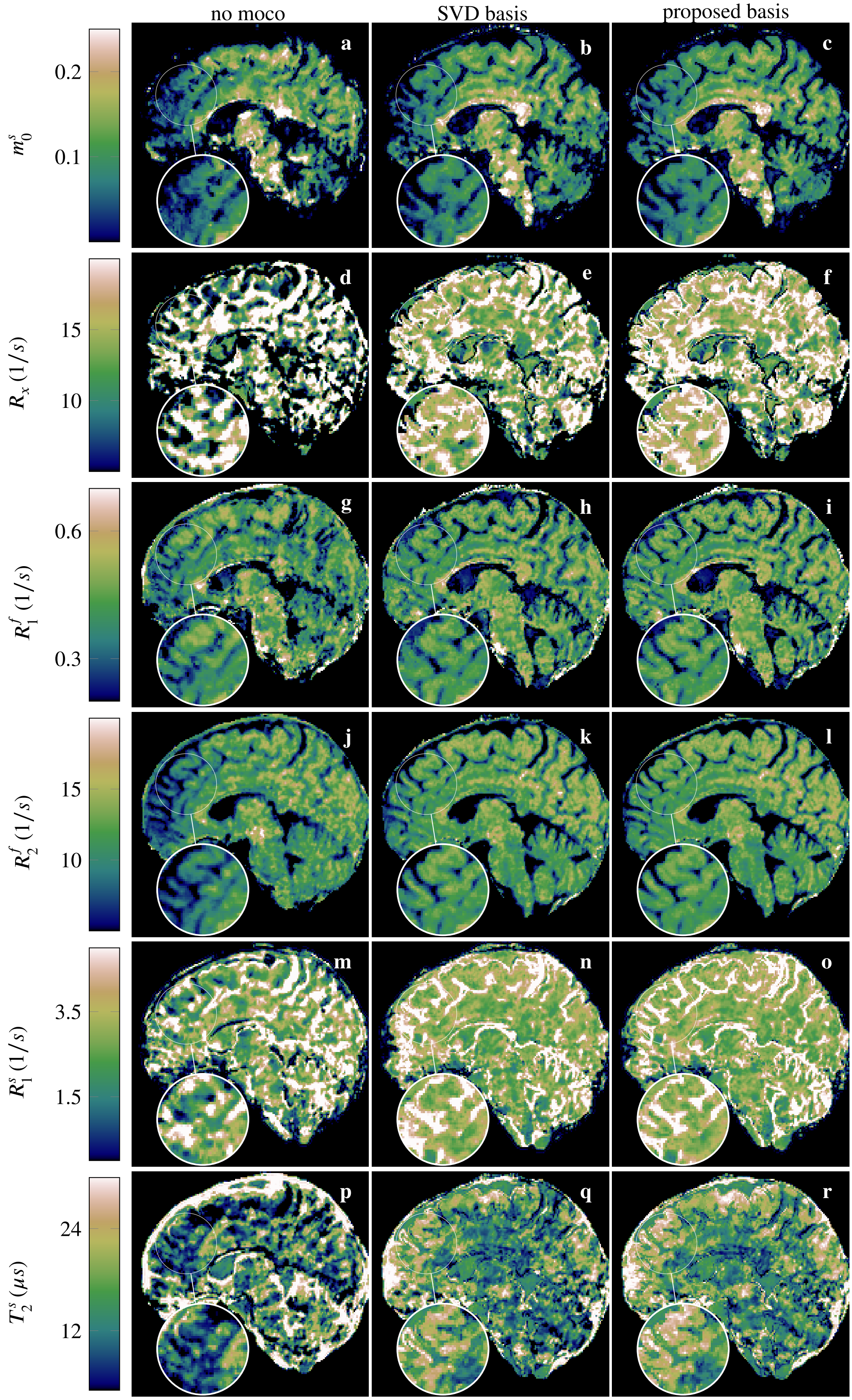

FIGURE S6 Sagittal view of qMT parameters maps in presence of strong motion caused by voluntary movement of the participant (Supporting Fig. S7). Motion correction with the original SVD basis substantially reduced motion artifacts, which are further reduced when using the proposed contrast-optimized basis.

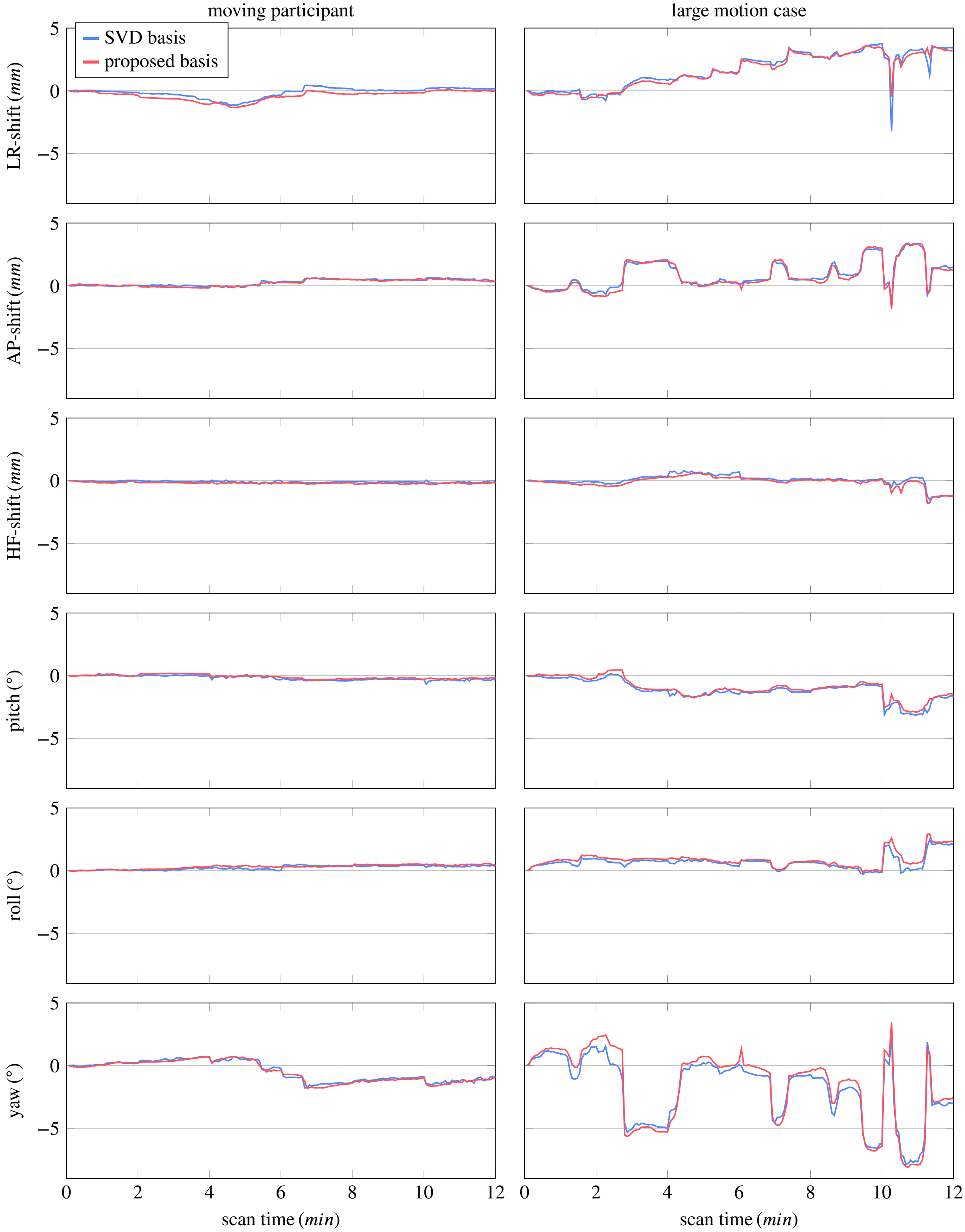

FIGURE S7 Motion parameters estimated using the SVD basis [8] and the proposed contrast-optimized basis in case of mild motion ("moving" participant), and in presence of large motion (motion score of 6.35 mm and 5.85 mm for the proposed contrast-optimized basis and the SVD basis respectively), which led to substantial degradation of the parametric maps, partially mitigated by the motion correction methods (Supporting Fig. S6).
